# Supplementary material for: Multi-bioinspired electronic skins with on-demand adhesion and opto-electronic synergistic display capabilities
Source: Innovation (Camb). 2025 Mar 12;6(5):100877. doi: 10.1016/j.xinn.2025.100877 (PMC12105526; doi:10.1016/j.xinn.2025.100877)
Supplement: Document S1. Figures S1–S13 and supplemental materials and methods [file mmc1.pdf]

The Innovation, Volume *6*

Supplemental Information

Multi-bioinspired electronic skins with on-demand adhesion and opto-electronic synergistic display capabilities

Wenzhao Li, Jinbo Li, Xiaoya Ding, Qitao Tan, Weijian Sun, Puxiang Lai, and Yuanjin Zhao

Supplemental information

Multi-bioinspired electronic skins with on-demand adhesion and opto-electronic synergistic display capabilities

Wenzhao Li [1,2], Jinbo Li [3], Xiaoya Ding [3], Qitao Tan[2, 4], Weijian Sun [1,5,*], Puxiang Lai [2, 4, 6, *], Yuanjin Zhao[1,3,*]

[1] Department of Rheumatology and Immunology, Nanjing Drum Tower Hospital, School of Biological Science and Medical Engineering, Southeast University, Nanjing 210096, China

[2] Department of Biomedical Engineering, The Hong Kong Polytechnic University, Hong Kong SAR 999077, China

[3] Wenzhou Institute, University of Chinese Academy of Sciences, Wenzhou, Zhejiang 325001, China

[4] Research Institute for Sports Science and Technology, The Hong Kong Polytechnic University, Hong Kong SAR 999077, China

[5] Department of Gastrointestinal Surgery, The First Affiliated Hospital, Wenzhou Medical University, Wenzhou 325035, China

[6] Joint Research Center for Biosensing and Precision Theranostics, The Hong Kong Polytechnic University, Hong Kong SAR 999077, China

Correspondence: fame198288@126.com (W. S.); puxiang.lai@polyu.eu.hk (P. L.); yjzhao@seu.edu.cn (Y. Z.)

1. Experimental section

Mechanical performance test

In the tensile tests, the tension started from the original length to break. While in the cyclic tests, strains of hydrogels were cycled between 0-100% for tension, for a total of 500 cycles. Breaking strength and strain are defined as the strain and strain at break.

Stress is the ratio of tension to the original cross-sectional area and strain is the ratio of elongation to original length.

Photothermal performance demonstration

The OE-skin was cut into 2×2 cm^2 size and adhered to pig skin. It was irradiated with 808 nm NIR laser of the same shape and size as the OE-skin. The power density of NIR is defined as the ratio of power to spot area. The OE-skin was irradiated with 0.25, 0.5, 0.75 and 1 $W\cdot cm^{-2}$ NIR. The temperature was recorded. In the photothermal cycling experiment, NIR was switched between 3 min on and 1 min off, repeating 4 cycles.

Shrinking and recovering of the hemispherical array

The hemispherical array was heterogeneously loaded with the fluorescent dye Rhodamine B. The OE-skin was attached to a transparent substrate. The hemispherical fluorescent area was recorded as S_0. Then the OE-skin was heated to 45°C using NIR and kept for 1 min. At this time, the hemispherical structure shrunk and separated from the substrate. The fluorescent area S was recorded again. The NIR was turned off, the shape of the hemispherical array was restored, and the area S was recorded again. The contraction rate was equal to S/S_0. The area S was measured by ImageJ.

Adhesion experiments

OE-skin was attached to pig skin, heated to 45°C using NIR and maintained at a pre-pressure of 10 kPa for 1 min, which was recorded as the adhesive state. When detached on demand, NIR was used to heat up to 45°C and maintained for 1 minute until the adhesion failed, which was recorded as the non-adhesive state. In tensile and shearing adhesion tests, separation was performed from the directions perpendicular and parallel to the OE-skin respectively until the adhesion broke. At this time, stress was recorded as the ratio of adhesion force to adhesion area. Strength is the maximum value of stress. Adhesion energy is the energy required for interface separation in

tensile adhesion experiments, which is the integral of adhesion force and displacement image area.

In Vitro cytocompatibility assay

3T3 cells were planted in 96-well plates as 1500 per well. The control group was cultured with DMEM medium, and the experimental group was cultured with 0.2 $g \cdot mL^{-1}$ OE-skin according to ISO 10993. The cells were incubated in a carbon dioxide incubator at 37°C for 72 h. The cells were tested according to the instructions of CCK-8 and live/dead staining kit at 24 h, 48 h and 72 h.

In Vitro blood compatibility assay

Fresh blood from rats was taken and centrifuged at 3000 rpm for 10 minutes to purify red blood cells. The blood was resuspended in 1×PBS and the above process was repeated three times. Finally, a red blood cell suspension (1% v/v) was prepared using PBS. 1, 5, 10, 50 and 100 $mg \cdot mL^{-1}$ of OE-skin were incubated with the above red blood cell suspension at 37 °C for 3 hours. Afterwards, the resulting liquid was centrifuged at 3000 rpm for 10 minutes to remove the precipitate. The absorbance in the supernatant was then measured within 540 nm. Red blood cells interacting with deionized water were used as positive controls and recorded as a hemolysis rate of 100%. Red blood cell suspensions in PBS were used as negative controls and recorded as a hemolysis rate of 0%.

In vivo animal experiment

Six male BALB/C nude mice aged 6 weeks were selected. They were adhered to the back of the nude mice. The adhesion and detachment processes were the same as above. They were allowed to move freely for one hour during adhesion. Commercial tape was used as a control and the adhesion was also maintained for one hour. The final state picture was taken ten minutes after detachment. The *in-situ* skin samples were subjected to histological analysis using H&E staining. All animal experiments were conducted in strict accordance with the recommendations in the Guide to the

Care and Use of Experimental Animals. The Animal Experiment Ethics Committee of Wenzhou Institute of University of Chinese Academy of Sciences reviewed and approved all animal care and experimental protocols. The approval number is WIUCAS24081502.

Physiological information monitoring

For the response curve of optical and electrical signals to temperature, OE-skin was attached to the surface of pig skin, and then the pig skin was placed on a hot plate to heat it, and the changes in resistance and spectral reflection peaks were recorded. For the response curve of optical and electrical signals to strain, the patch was attached to the regular rectangular Ecoflex surface to simulate biological tissue. Ecoflex has higher elasticity than pig skin, and pig skin will undergo irreversible deformation under large strains, affecting the experiment. The resistance and reflection peak values were read as they were stretched. The resistance value in the initial state was recorded as R_0, and the change was recorded as ΔR. The change in the reflection peak was recorded as shift value. For repeated motion monitoring, the patch was attached to the index finger, wrist, elbow and knee joints, and recorded continuously. All resistances were recorded using a desktop digital multimeter. The spectrum was recorded using a spectrometer. All data sampling points were read after stabilization.

Signal processing of OE-skin by neural network

A fully connected neural network (FCNN) model was used to perform regression analysis on the relationship between temperature, strain, ΔR/R0 and shift value. The model consists of an input layer, a hidden layer and an output layer, and information flows unidirectionally from the input layer to the output layer. The input layer contains four neurons, representing the four input variables mentioned above. The hidden layer contains two layers, the first hidden layer contains five neurons, and the second hidden layer contains three neurons. These layers are fully connected, that is, each neuron in each layer is connected to each neuron in the next layer. The output

layer contains two neurons, representing the two target variables to be predicted. Before training the neural network, the input data is normalized. Each data point contains four variables, and a total of 600 variables were collected, generating 150 data points. Randomly divide two-thirds of these data points into training sets and the rest into test sets. The epoch is 100 iterations. Loss function based on the mean square error is used to measure the difference between the predicted value and the actual value.

Characterization

The micro- and nanostructures were observed by SEM (SU8010, HITACHI, Japan). The observed optical and fluorescence microscopic images were obtained by stereomicroscopy (Olympus BX51, Tokyo, Japan) and inverted fluorescence microscopy (ZEISS Axio Vert. A1, Germany). Micro-CT images were obtained by micro-computed tomography (SkyScan 1176, Bruker, Germany). Rheological properties were tested by a rotational TA rheometer (DHR-2). Electrical resistance was tested by a benchtop digital multimeter (DMM6500, Keithley, USA). The absorption spectrum of the solution was measured by a multi-mode microplate reader (Varioskan LUX, ThermoFisher, USA). Mechanical testing was performed with an electronic universal material testing machine (5944, Instron, USA). 808 nm NIR was generated by a laser (DS3-808 nm -10 W BWT China). The temperature was recorded by an infrared thermometer (E5-XT, FLIR, USA). The reflectance spectra were taken by an optical microscope (Olympus, BX51) equipped with a fiber optic spectrometer (Ocean Optics, USB2000-FLG).

Statistical analysis

Unless specifically mentioned, the whole data were expressed as mean ± standard deviations in multiple repeated groups. The standard deviation is represented by the error bars. For direct comparisons between two groups, Student's t-test was employed. When more than two groups were involved, one-way ANOVA was utilized. The differences were considered statistically significant if $*p < 0.05$, $**p < 0.01$, $***p <$

0.001 or ****$p < 0.0001$. Sample size (n) is detailed in the specific figure legends. All statistical analysis was conducted using SPSS software.

2. Supplemental Figures

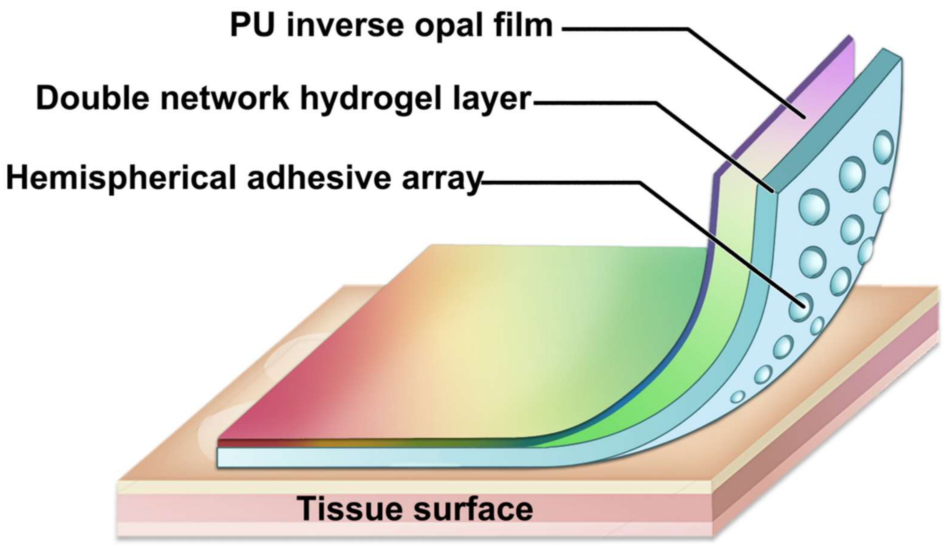

Figure S1. OE-skin includes PU film and hydrogel layer. There is an adhesive array on the hydrogel layer.

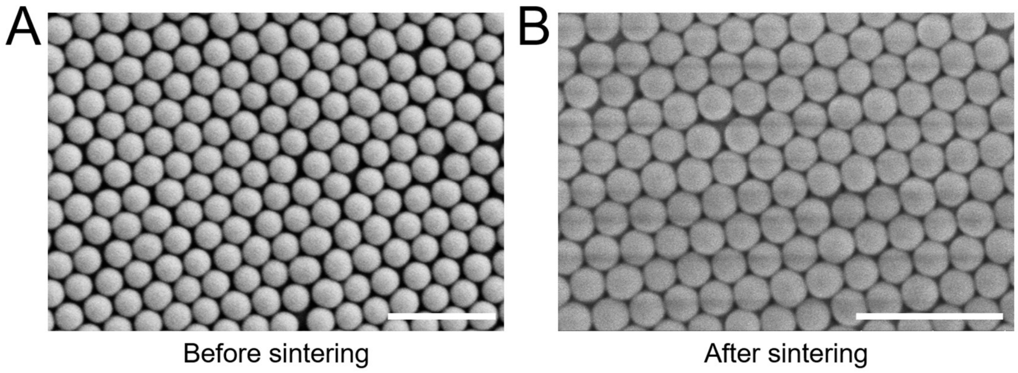

Figure S2. Comparison of SiO_2 template before and after sintering. The particles are more closely spaced after sintering. (A) Before sintering. (B) After sintering. Scale bars are 1 μm.

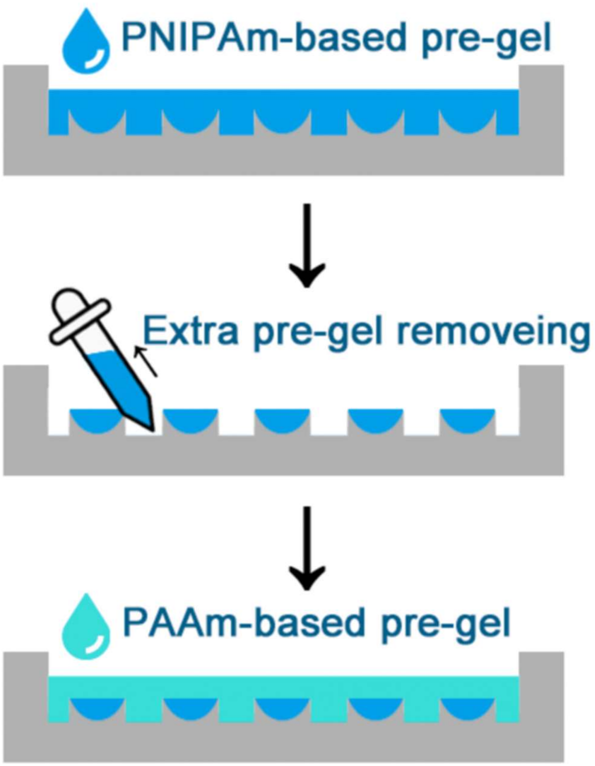

Figure S3. Detailed schematic diagram of the stepwise template infusion method. Two pre-gel solutions of hydrogels were infused separately and cross-linked to obtain a heterogeneous structure.

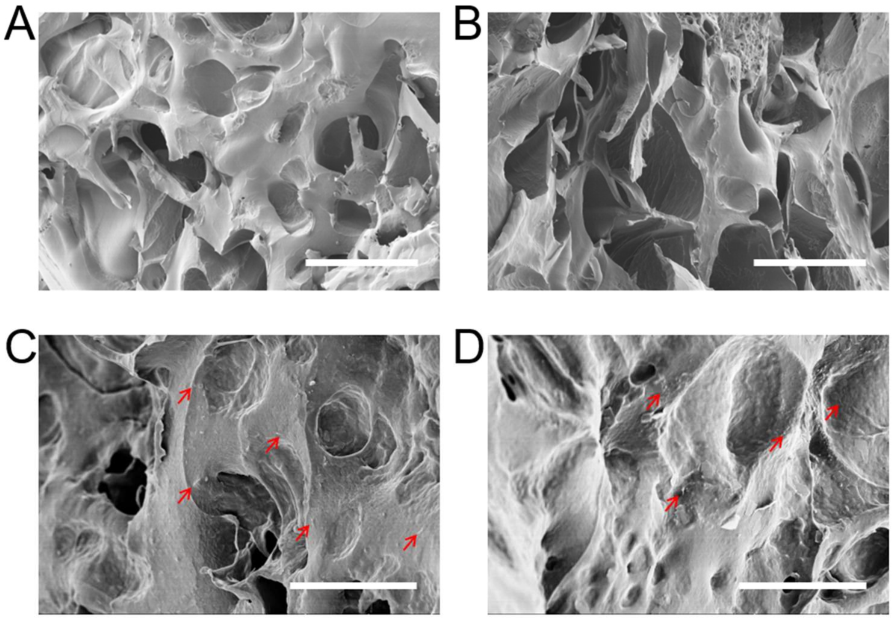

Figure S4. SEM images of hydrogel pores. (A-B) PAAm (left) and PNIPAm (right) double network hydrogels have rich porous structures. (C-D) CNTs are dispersed in the pores of PAAm (left) and PNIPAm (right) double network hydrogels. Scale bars are 100 μm in (A) and (B), as well as 10 μm in (C) and (D).

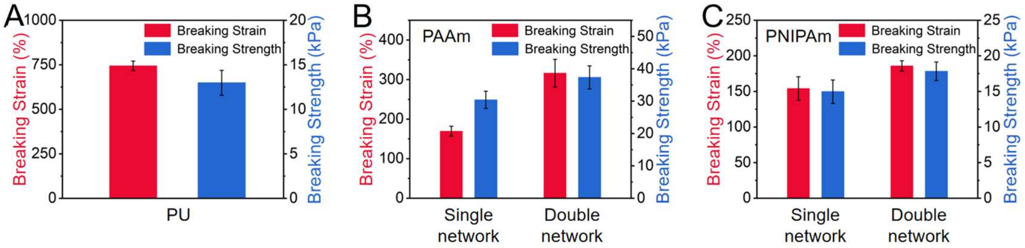

Figure S5. The mechanical properties of PU film, single and double network hydrogels. (A) PU. (B) Single and double network PAAm. (C) Single and double network PNIPAm. Double network hydrogels generally have better mechanical properties. n=3 for each statistic.

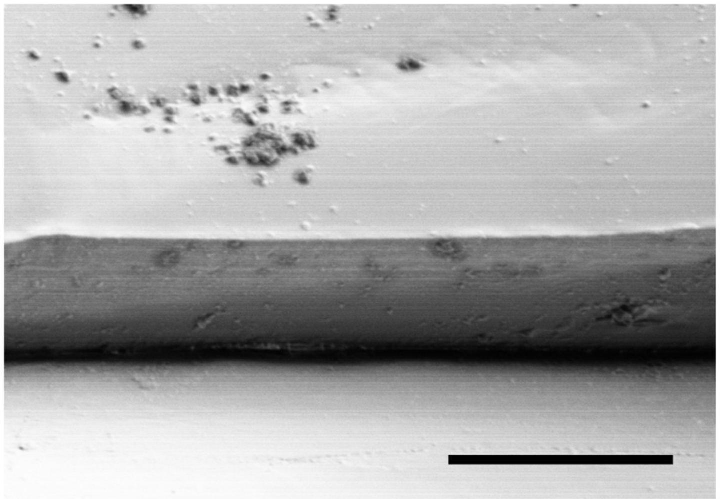

Figure S6. The interface between PU film and hydrogel layer is tightly coupled. The scale bar is 20 μm.

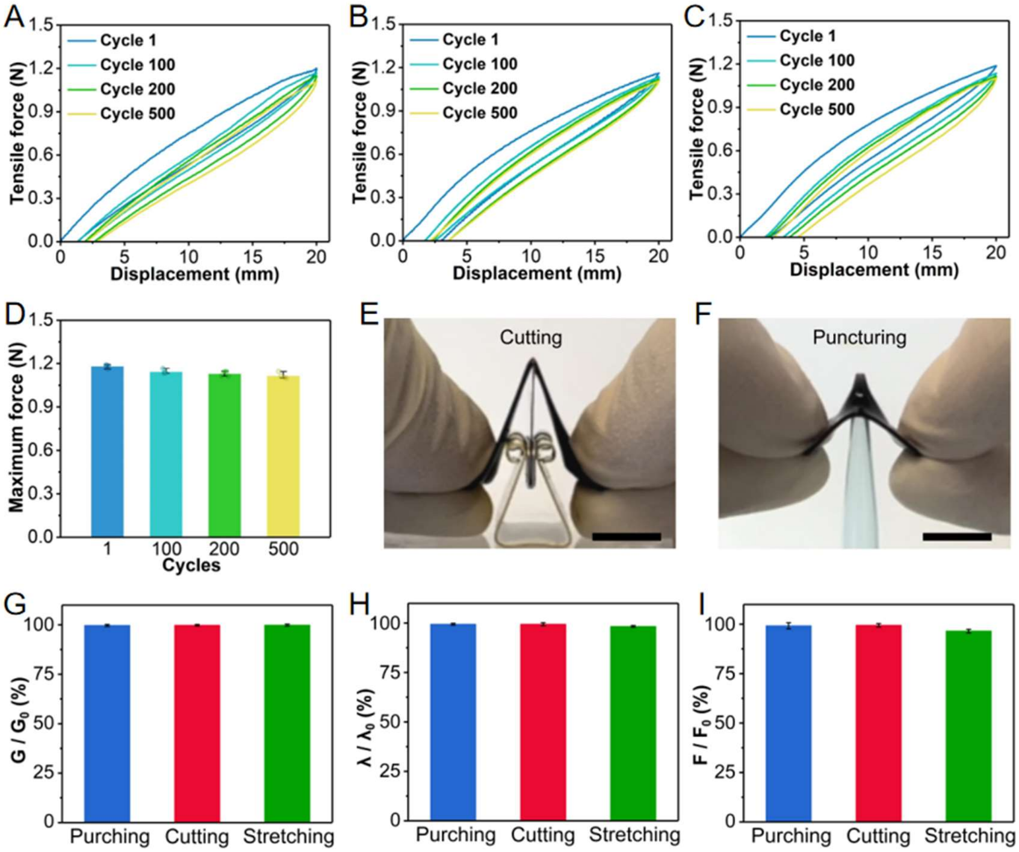

Figure S7. Mechanical robustness of OE-skin. (A-D) OE-skin has mechanical robustness in cyclic tensile tests. Additional parallel experiments are in (A-C), and statistical analysis of the maximum tensile force is in (D), n=4. (E-F) OE-skin has good anti-breaking performance. The cutting test is in (E), and the puncturing test is in (F). Scale bars are 1 cm. (G-I) The electrical, optical, and mechanical performance of OE-skin after puncturing, cutting, and cyclic stretching. The changes in conductivity (G), reflection peak (λ), and tensile force (F) are shown in (G), (H), and (I), respectively, n=3.

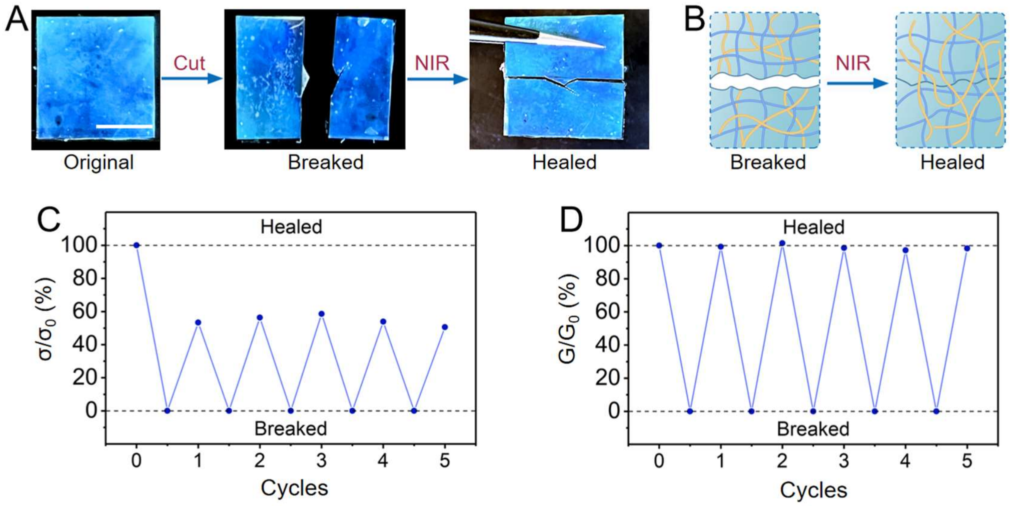

Figure S8. NIR-controlled healing properties of OE-skin. (A) Physical images of the OE-skin being broken and repaired by NIR. (B) Schematic diagram of the healing process. (C) Changes in fracture strength σ during in five breaked-healed cycles. (D) Corresponding conductivity G changes. Scale bars are 1 cm.

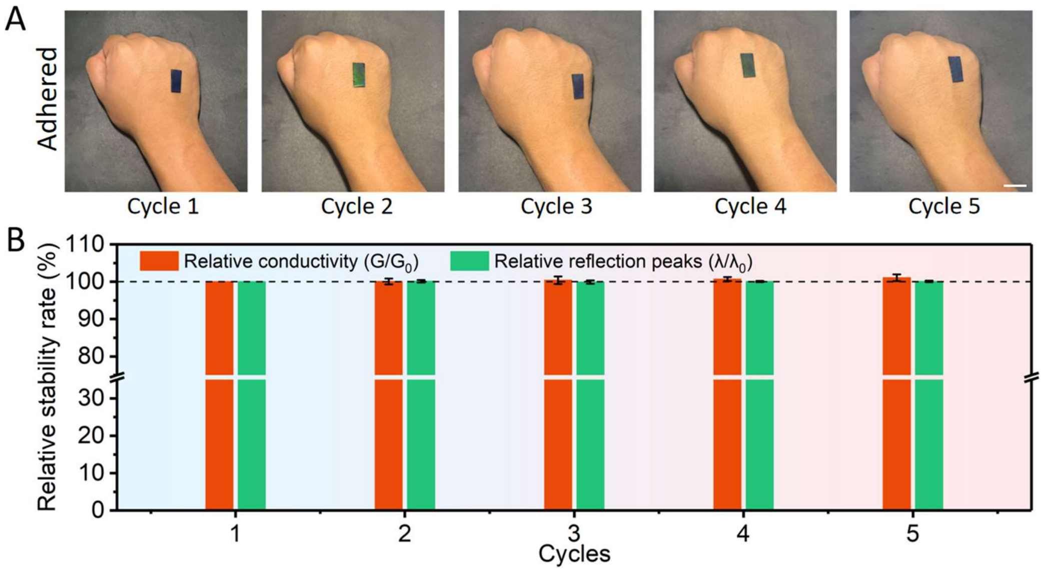

Figure S9. *In vivo* adhesion cycles and corresponding performance stability. (A) Five cycles of adhesion and repositioning of OE-skin on the human skin. The adhesion remained robust without any discomfort. The scale bar is 1 cm. (B) The optical and electrical properties during 5 cycles.

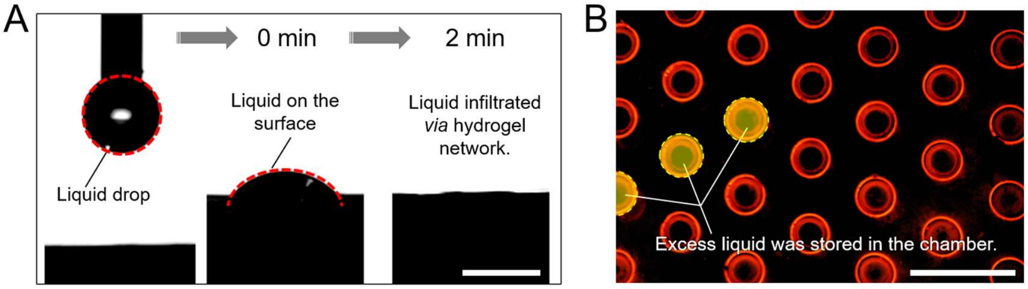

Figure S10. Permeability and water resistance of OE-skin. (A) Droplets used to simulate sweat were applied to the non-array area of OE-skin. The droplets quickly penetrated the hydrogel network within 2 minutes, demonstrating the permeability of OE-skin to sweat. (B) Fluorescence image of interfacial water trapped by the adhesive array. The interfacial water is dyed red by fluorescent dye. Scale bars are 1 mm in (A) and 2.5 mm in (B).

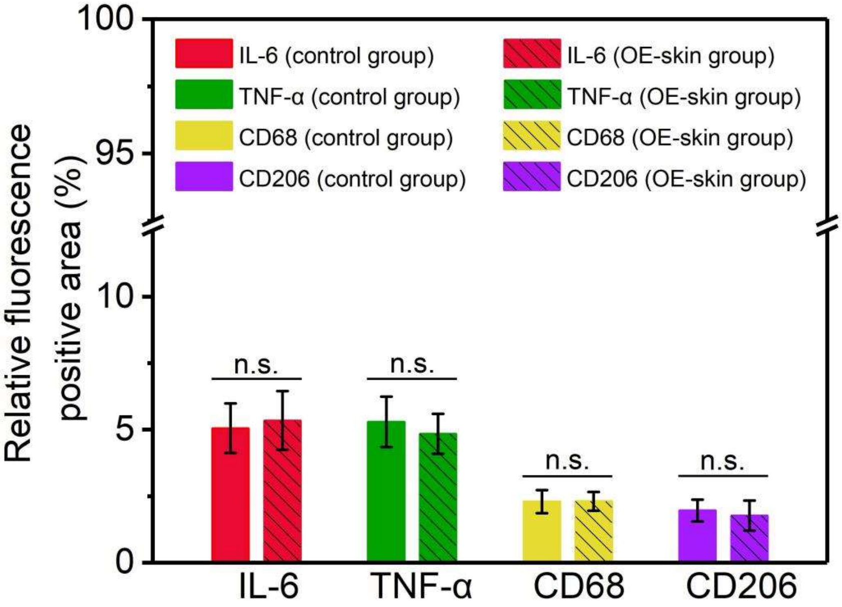

Figure S11. Relative fluorescence positive area in immunofluorescence sections of inflammation and immunity markers. There was no statistical difference between OE-skin groups and the control group, further indicating that the long-term tissue compatibility of OE-skin.

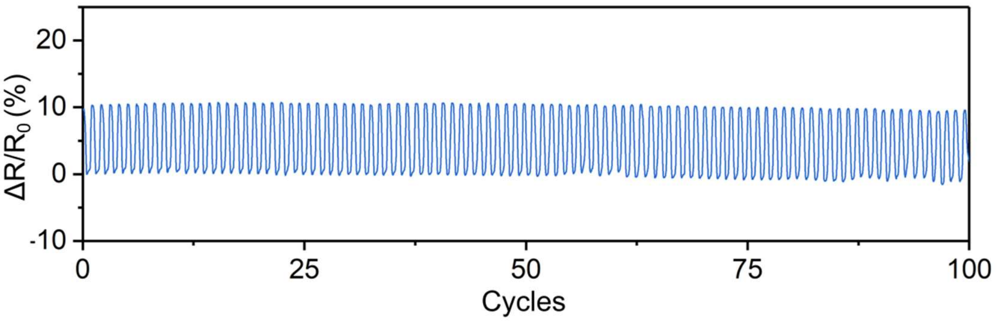

Figure S12. OE-skin collects 100 cycles of motion signals from the wrist.

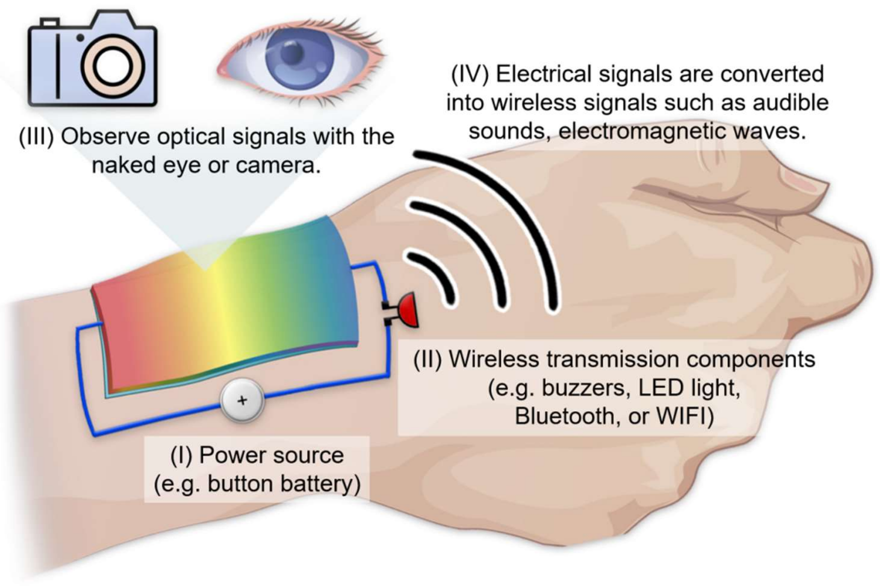

Figure S13. The integration of OE-skin into a fully wireless electronic skin is illustrated in a simple and cost-effective manner. (I) Miniaturized power source. (II) Miniaturized wireless transmission components. (III) Optical signals can be observed visually. (IV) Electrical signals (resistance changes) are converted into other wireless signals, such as audible sound and electromagnetic waves. Audible sound can be directly perceived by the human ear.
