# Supplementary material for: Multi-bioinspired electronic skins with on-demand adhesion and opto-electronic synergistic display capabilities
Source: Innovation (Camb). 2025 Mar 12;6(5):100877. doi: 10.1016/j.xinn.2025.100877 (PMC12105526; doi:10.1016/j.xinn.2025.100877)
Supplement: Document S2. Article plus supplemental information [file mmc7.pdf]

OPEN ACCESS

Multi-bioinspired electronic skins with on-demand adhesion and opto-electronic synergistic display capabilities

Wenzhao Li,[1,2] Jinbo Li,[3] Xiaoya Ding,[3] Qitao Tan,[2,4] Weijian Sun,[1,5,*] Puxiang Lai,[2,4,6,*] and Yuanjin Zhao[1,3,*]

*Correspondence: fame198288@126.com (W.S.); puxiang.lai@polyu.eu.hk (P.L.); yjzhao@seu.edu.cn (Y.Z.)

Received: August 19, 2024; Accepted: March 7, 2025; Published Online: March 12, 2025; https://doi.org/10.1016/j.xinn.2025.100877

© 2025 The Authors. Published by Elsevier Inc. on behalf of Youth Innovation Co., Ltd. This is an open access article under the CC BY license (http://creativecommons.org/licenses/by/4.0/).

GRAPHICAL ABSTRACT

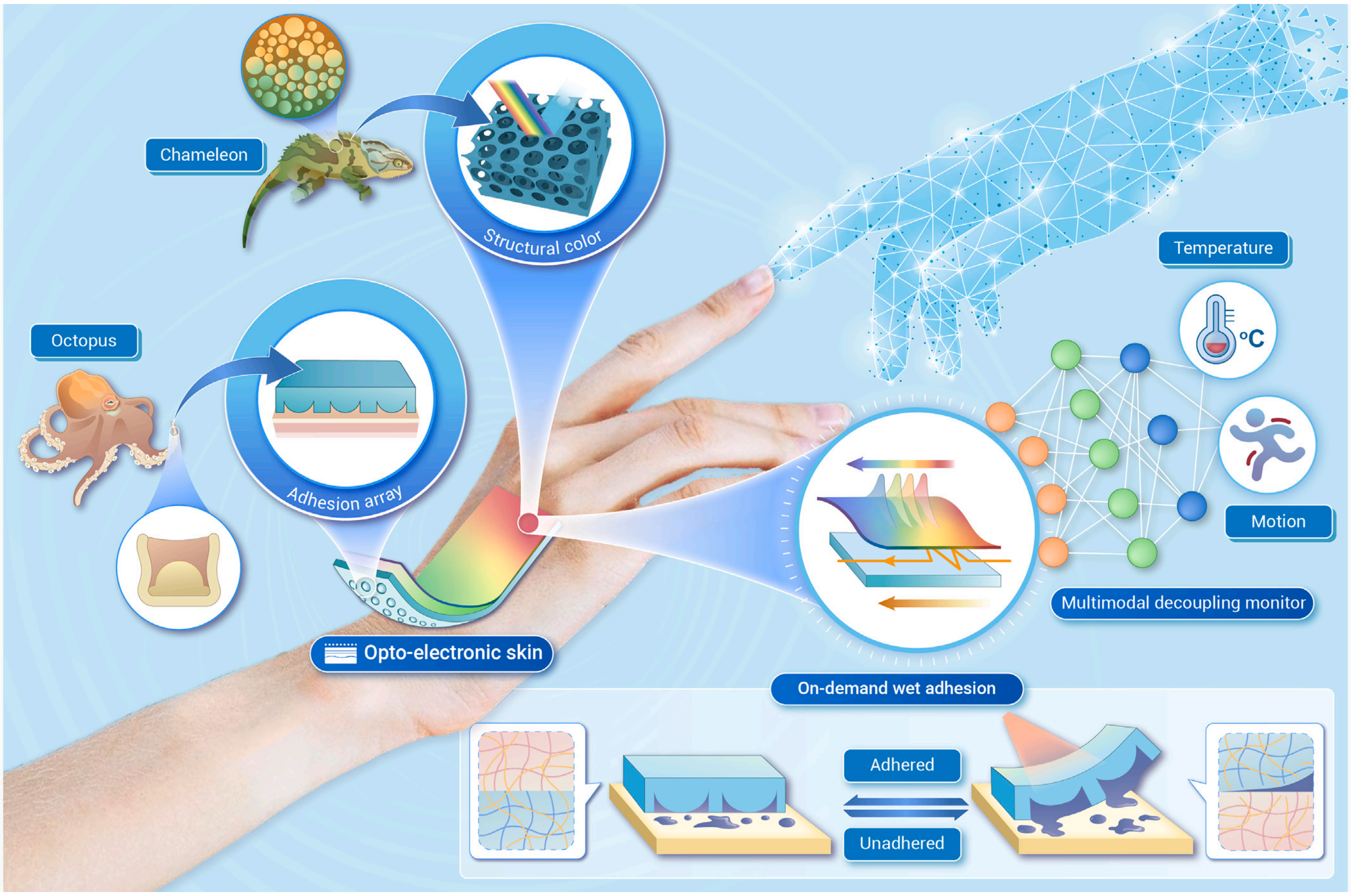

PUBLIC SUMMARY

- The bioinspired flexible opto-electronic skin (OE-skin) mimics organism nano-micro structures.
- The OE-skin enables NIR-controlled on-demand adhesion with safe robustness at biological interfaces.
- Validated across cellular to organism levels, it ensures biocompatibility for prolonged wearable use.
- The OE-skin achieves *in vivo* multimodal signal acquisition, decoupling dual physiological signals.
- The OE-skin integrates microelectronics and AI for physiological monitoring and human-computer interactions.

Multi-bioinspired electronic skins with on-demand adhesion and opto-electronic synergistic display capabilities

Wenzhao Li,[1,2] Jinbo Li,[3] Xiaoya Ding,[3] Qitao Tan,[2,4] Weijian Sun,[1,5,*] Puxiang Lai,[2,4,6,*] and Yuanjin Zhao[1,3,*]

[1]Department of Rheumatology and Immunology, Nanjing Drum Tower Hospital, School of Biological Science and Medical Engineering, Southeast University, Nanjing 210096, China
[2]Department of Biomedical Engineering, The Hong Kong Polytechnic University, Hong Kong SAR 999077, China
[3]Wenzhou Institute, University of Chinese Academy of Sciences, Wenzhou, Zhejiang 325001, China
[4]Research Institute for Sports Science and Technology, The Hong Kong Polytechnic University, Hong Kong SAR 999077, China
[5]Department of Gastrointestinal Surgery, The First Affiliated Hospital, Wenzhou Medical University, Wenzhou 325035, China
[6]Joint Research Center for Biosensing and Precision Theranostics, The Hong Kong Polytechnic University, Hong Kong SAR 999077, China
*Correspondence: fame198288@126.com (W.S.); puxiang.lai@polyu.eu.hk (P.L.); yjzhao@seu.edu.cn (Y.Z.)

Received: August 19, 2024; Accepted: March 7, 2025; Published Online: March 12, 2025; https://doi.org/10.1016/j.xinn.2025.100877

© 2025 The Authors. Published by Elsevier Inc. on behalf of Youth Innovation Co., Ltd. This is an open access article under the CC BY license (http://creativecommons.org/licenses/by/4.0/).

Citation: Li W., Li J., Ding X., et al., (2025). Multi-bioinspired electronic skins with on-demand adhesion and opto-electronic synergistic display capabilities. The Innovation **6(5)**, 100877.

Flexible electronic skins hold great promise for biomedical applications, although challenges remain in achieving controllable interactions with the biological interface and accurate signal collection. Inspired by octopuses and chameleons, we propose a novel electronic skin paradigm with on-demand adhesion and opto-electronic synergistic display capabilities. Our electronic skins are composed of a stretchable polyurethane (PU) inverse opal film integrated with a carbon nanotube (CNT)-hybridized polyacrylamide (PAAm)-gelatin double-network-hydrogel conductive flexible substrate and a temperature-responsive poly(N-isopropylacrylamide) (PNIPAm) octopus-inspired hemispherical adhesive array. The device's CNT hybrid double-network provides robust and sensitive monitoring of temperature and motion. Meanwhile, its flexible PU layer with an inverse opal structure allows for visual motion color sensing. Integrated neural network processing ensures accurate, wide-range, and independent multimodal display. Additionally, the integration of the photothermal effect of CNTs and the temperature-sensitive octopus-inspired PNIPAm adhesive array enables on-demand adhesion. The sensing and adhesion demonstrations *ex vivo* and *in vivo* showcase the proposed flexible electronic skin's inspirational design and functional utilities. The potential applications of such a versatile device are vast, ranging from healthcare to human-machine interactions.

INTRODUCTION

Flexible electronic skins have profound implications and broad prospects in biomedicine, including health monitoring, human-machine interactions, intelligent prosthetics, etc.[1–5] The application of electronic skins typically requires designing and constructing flexible conductive materials,[6–8] followed by the skins interacting with the biological interface and the monitoring of physiological signals.[9–13] These expectations pose severe challenges for interactions with the biological interface and the execution of the electrical functionality[14–17]: current adhesion systems will suffer from deficiencies in controllability and robustness, resulting in intractable regulation of adhesion and detachment behaviors.[18–23] In addition, in terms of electrical functionality, most current flexible electronics can only detect a single modality of electronic signals with low accuracy but high interference.[24–26] Therefore, there is an urgent need to develop a new type of flexible electronics with advanced adhesion behavior and precise multimodal synergistic capability.

Here, inspired by multiple mechanisms of octopus adhesion and chameleon color change, we propose an innovative paradigm of electronic skins with on-demand adhesion and opto-electronic synergistic information display capabilities, as shown in Figure 1. In nature, octopuses achieve reversible adhesion by manipulating the motions of the hemispherical structures in their suckers, adapting to diverse demands.[27–30] On the other hand, chameleons can rapidly alter their skin color to adapt to the environment due to the structural color generated by the nano-gratings in their pigment cells.[31–33] Mimicking these phenomena, functional materials with unique adhesive and optical properties possess potential values in different areas. In particular, by further synergizing the optical and electrical information with signal processing means, such as neural networks, an excellent display of uncoupled multimodal physiological signals is expected to be achieved.[34,35] Therefore, mimicking these bioinspired features reveals insights into a novel class of innovative and superior intelligent, flexible electronic devices.

In this paper, we employ a carbon nanotube (CNT)-hybridized polyacrylamide (PAAm)-gelatin double-network hydrogel to construct a conductive flexible substrate and endow it with a temperature-responsive poly(N-isopropylacrylamide) (PNIPAm) octopus-inspired hemispherical adhesive array. We further integrate it with a flexible polyurethane (PU) inverse opal film to design the opto-electronic skin (OE-skin), which allows for satisfying controllable and robust adhesion as well as multimodal physiological information monitoring on the biological interface. Owing to the CNT and PAAm network, this electronic skin has superior robustness and sensitive monitoring ability for temperature and motion. Simultaneously, the photothermal effect of CNTs and the temperature sensitivity between the octopus-inspired PNIPAm adhesive array synergize to achieve on-demand adhesion behavior. More attractively, benefiting from the excellent flexibility of the PU layer and the inverse opal structure, this device can also realize visual color sensing for motions. By further processing the opto-electronic synergistic signals through a neural network, we can achieve accurate, independent, and interference-free temperature and motion display. These features indicate that the proposed flexible electronic skin has an advanced design concept and practical functionality.

MATERIALS AND METHODS

Materials

Gelatin from porcine skin (~300 g Bloom), acrylamide, N-isopropylacrylamide, and Bis-MBAA were purchased from Sigma-Aldrich (USA). HF, ammonium persulfate, Rhodamine B, and tetramethylethylenediamine (TEMED) were purchased from Macklin (China). PU was purchased from Zhejiang Huafon Spandex. SiO_2 nanoparticles were self-prepared through the Stöber method. 3T3 cells and DMEM medium were purchased from ScienCell (China). The Cell Counting Kit-8 (CCK-8) and live/dead cell viability kit were purchased from Thermo Fisher Scientific (USA). CNTs were obtained from XFNANO (China). Ecoflex was purchased from Smooth-On (USA). All other reagents used in the experiments were of analytical grade and used directly.

Preparation of PU inverse opal films

First, several batches of SiO_2 nanospheres with diameters ranging from 220 to 320 nm were selected through differential centrifugation. These nanospheres were dispersed in ethanol to form a 20% w/v suspension using ultrasonic treatment. The suspension was then uniformly coated onto glass slides via self-assembly, allowing the ethanol to evaporate naturally over the course of 1 h. Subsequently, the primary templates were calcined at 600°C for 9 h, resulting in the opal templates.

Next, a PU solution was prepared by dissolving PU at a concentration of 20% w/v in DMF. This solution was stirred at 120 rpm in a 60°C water bath for 24 h until homogeneous. The cooled PU solution was then poured into the opal templates and spin coated at 300 rpm for 100 s. The coated templates were placed on a hot plate at 80°C to gradually cure until the solvent completely evaporated, yielding a hybrid template. Finally, the films were immersed in 40% w/v HF to etch away the templates, resulting in the formation of PU inverse opal films. Distilled water was used to wash away any residue.

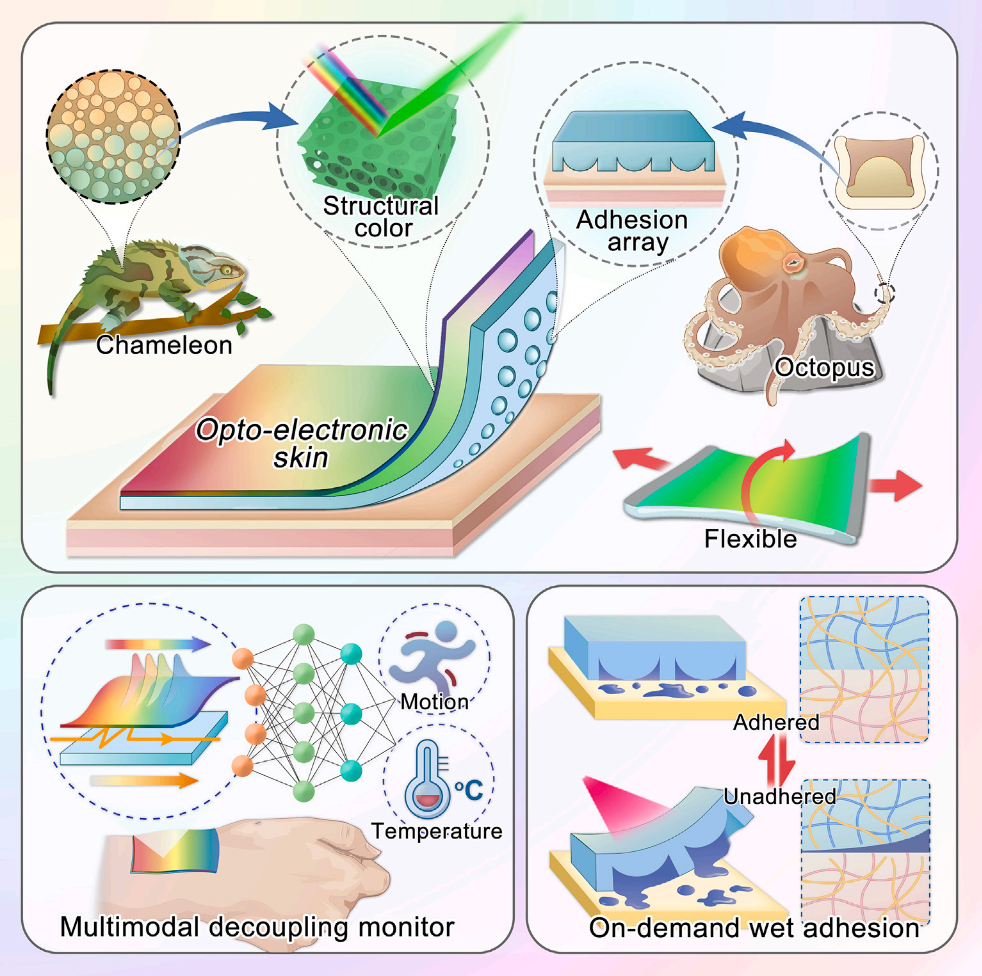

Figure 1. Schematic diagram of multi-bioinspired OE-skins The flexible device is composed of inverse opal film integrated with conductive double-network hydrogel substrate and temperature-responsive, octopus-inspired hemispherical adhesive array. It has on-demand adhesion and opto-electronic synergistic display capabilities.

Preparation of the single-network hydrogels

The single-network hydrogel was formed by free radical polymerization. 20% w/v AAm or NIPAm monomer, 0.5% w/v initiator potassium persulfate, and 0.1% w/v crosslinker Bis-MBAA were dissolved in ultrapure water and homogenized by stirring at 40°C to obtain a pre-gel. To initiate free radical polymerization, 1 μL mL^{-1} of TEMED was added to the above solution. The single-network hydrogel of PAAm or PNIPAm was formed by standing in a nitrogen atmosphere at room temperature for 10 min.

Preparation of the double-network hydrogels

The double-network hydrogel was similar to the single network, and its covalent network was formed by free radical polymerization. 20% w/v AAm or NIPAm monomer, 10% w/v gelatin, 0.5% w/v initiator potassium persulfate, 0.1% w/v crosslinker Bis-MBAA, and 1% w/v CNTs were dissolved in ultrapure water and homogenized by stirring at 40°C to obtain a pre-gel. To initiate free radical polymerization, 1 μL mL^{-1} of TEMED was added to the above solution. The single-network hydrogel of PAAm or PNIPAm was formed by standing in a nitrogen atmosphere at room temperature for 10 min.

Preparation of the OE-skin

First, the hydrogel layer was constructed using the stepwise template method. The pre-gel solution of double-network PNIPAm was filled into the hemispherical cavity of the mold under vacuum, and the excess solution was removed. The PNIPAm network was allowed to crosslink for 10 min. The pre-gel solution of double-network PAAm was then further infused to form a backing layer. Before crosslinking, the PU inverse opal film was covered on the back to form a coupling.

Other additional details are provided in the supplemental information.

RESULTS AND DISCUSSION

In the experiment, our OE-skins are composed of an elastic PU inverse opal film and a conductive double-network hydrogel layer. The hydrogel layer features an octopus-inspired hemispherical adhesive array (Figure S1). Initially, the PU inverse opal film with structural color was prepared using the opal template method (Figure 2A). This template was formed by the bottom-up self-assembly of SiO_2 nanospheres (Figure 2B). Precisely, the SiO_2 dispersion liquid was uniformly coated onto a substrate. As the solvent gradually evaporated, the SiO_2 formed a highly ordered, close-packed repeating sequence spatially. Further sintering was performed to enhance its connection (Figures S2A and S2B). Subsequently, the interval of this opal template was infiltrated with the PU via capillary forces, forming a hybrid template. The opal template was then etched away, resulting in a PU film with an inverse opal structure. Under scanning electron microscopy (SEM), a regular multilayer porous structure could be observed (Figure 2C).

The refractive index of the inverse opal structure exhibits periodic variations at nanoscale, resulting in photonic band-gap properties. Hence, light of specific frequencies is selectively reflected, which imparts the PU film with its brilliant and unique structural color. The diverse structural colors, corresponding to various wavelengths of reflection peaks, can be modulated by multiple factors. For normal incident light, the corresponding reflection peak follows Bragg's law:

$$\lambda = 1.633\, d \cdot n_{average.}$$

Therefore, a series of SiO_2 nanosphere opal templates with different interplanar spacing d were replicated. The corresponding PU inverse opal films with various reflection peaks λ can be achieved, displaying a rich array of colors, with constant refractive index n as shown in Figure 2D.

Furthermore, a heterogeneous hydrogel adhesive layer was simply prepared using a stepwise template infusion method (Figures 2E and S3) and coupled with the PU inverse opal film (Figure 2E), ultimately obtaining the OE-skin. The backing layer and adhesive array were composed of PAAm-based and PNIPAm-based double-network hydrogels, respectively (Figure S3). Microcomputed tomography (micro-CT) three-dimensional (3D) reconstruction results displayed an ordered and regular hemispherical adhesive array (Figure 2F). Stereoscopic fluorescence images further demonstrated the heterogeneity of the hemispheres and backing through different fluorescent staining, which were dyed red and green, respectively (Figure 2G). At the microscale, the hydrogels exhibited an abundant porous network structure (Figures S4A and S4B). Additionally, the doped CNTs could be observed to be dispersed within the network, as indicated by the arrows (Figures S4C and S4D). This microscopic uniformity ensures the robustness of the OE-skin. Benefiting from the toughening of the interpenetrating network and non-covalent interactions such as hydrogen bonding, the above double-network hydrogels exhibit superior mechanical properties compared to single networks. The elastic PU also shows high fracture strain and fracture strength, making them suitable for constructing flexible electronic devices (Figures S5A–S5C).

After the coupling process, the OE-skin exhibits more vivid structural colors compared to the isolated PU inverse opal film (Figure 2H), which is due to the CNTs providing a darker background that absorbs scattered light (Figure 2I).

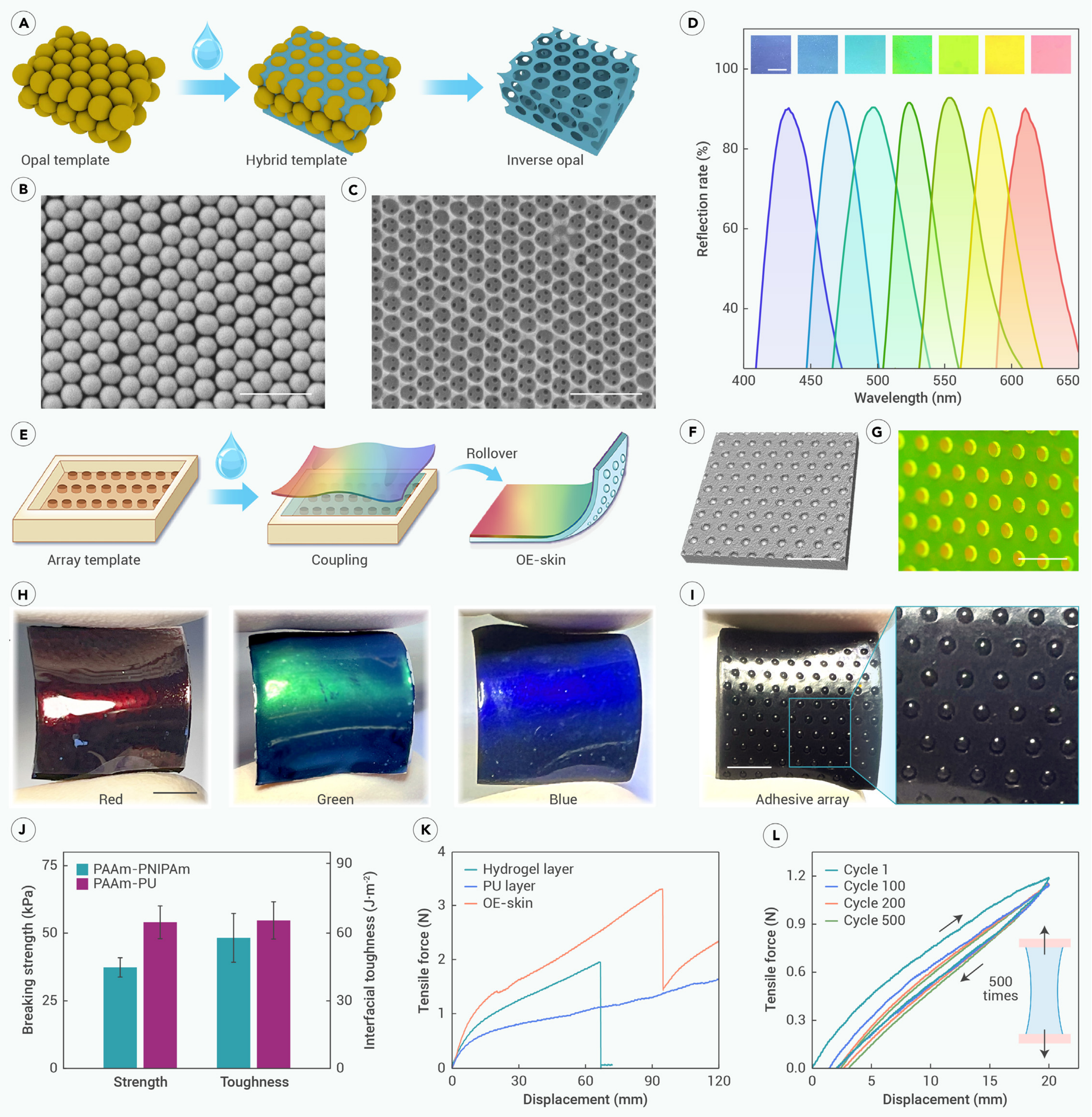

Figure 2. Preparation and characterization of OE-skin (A) Preparation of PU inverse opal film. PU is filled into the opal template self-assembled from SiO_2 nanospheres to obtain a hybrid template. Then, SiO_2 is etched away to finally obtain the inverse opal PU film. (B) SEM image of opal template self-assembled from SiO_2 nanospheres with an ordered structure. (C) SEM image of inverse opal structure. The structure is opposite that of the opal template and has ordered pores. (D) Reflective spectra of PU inverse opal film. Films of various colors can be obtained by controlling the parameters. (E) Stepwise template method of hydrogel layer and coupling of PU inverse opal film. Finally, the OE-skin is obtained. (F) Micro-CT image of OE-skin. Its surface has a hemispherical adhesive array. (G) Fluorescence image with heterogeneity. The hydrogel backing layer is dyed green, while the adhesive array is dyed red. (H) OE-skin with different structural colors. (I) Hydrogel with adhesive array layer. Its dark background makes the structural color more vivid. (J) Mechanical tests of firm coupled interface. n = 3. (K) Tensile fracture test. The coupling of PU film and hydrogel layer enhances the tensile performance. (L) Cyclic stretching test. The OE-skin shows stability in 500 cycles of tensile. Scale bars: 1 μm in (B) and (C) and 5 mm in (D) and (F)–(I).

The coupling between the hydrogel adhesive layer and the inverse opal film was maintained by the intermolecular forces and interfacial interlocking. With SEM, we could observe that the interface of the two layers was tightly bonded (Figure S6). A quantitative mechanical test of the coupling strength was carried out, including the PAAm-based hydrogel with PNIPAm-based hydrogels and the PAAm-based hydrogel with PU (Figure 2J). The breaking strengths both exceeded 30 kPa, which was over 1,000 times the weight of the OE-skin. The interfacial toughness also exceeded 50 J m^{-2}, demonstrating structural stability and difficulty in layer separation.

The tensile resistance of the OE-skin was also improved compared to the isolated hydrogel layer or PU film (Figure 2K). As the original length was set to 20 mm, the isolated hydrogel layer achieved a fracture elongation of over

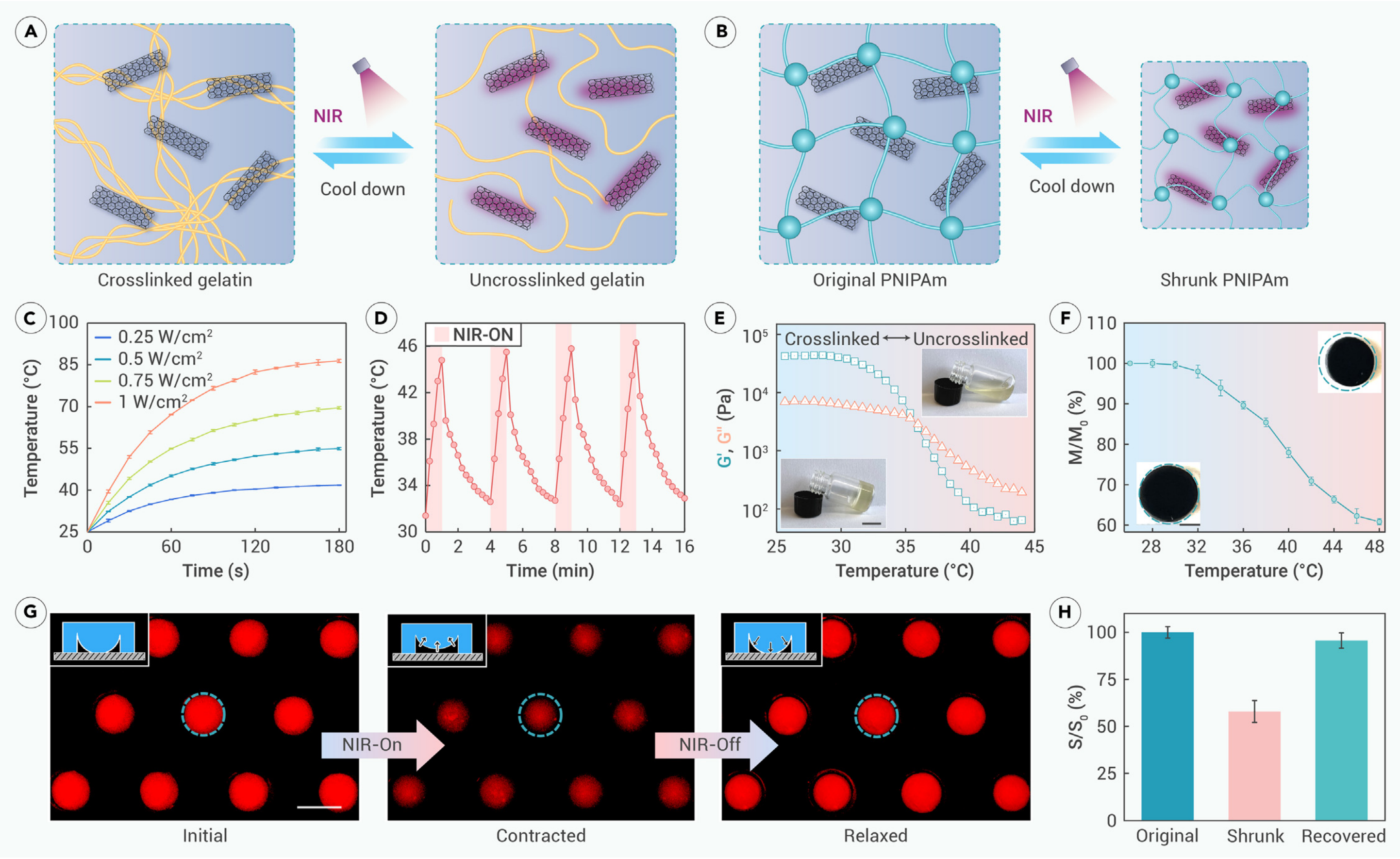

Figure 3. NIR-controlled behaviors of the OE-skins (A and B) Responsiveness of hydrogel network components under NIR irradiation. CNTs undergo photothermal conversion, and the temperature rise causes the uncrosslinking of gelatin and the shrinking of PNIPAm. (C) Temperature changing curve of OE-skin under different power densities of NIR. (D) Temperature cycling curve under periodic irradiations of NIR. (E) Rheological curve of isolated gelatin network, proving the uncrosslinking caused by temperature rise. (F) Change curve of PNIPAm-based hydrogel's mass *M* with temperature. Shrinkage occurs when the temperature rises. M_0 stands for initial mass. (G and H) Fluorescence images of the hemispherical array adhered to the substrate before and after NIR irradiation and the statistics of the change rate of their area *S*. *n* = 6. Scale bars: 1 cm in (E), 0.5 cm in (F), and 1 mm in (G).

300% (displacement > 60 mm), which fully meets the needs of flexible electronics. The coupled OE-skin showed an even higher fracture elongation of over 450% (displacement > 90 mm), likely due to the effect of hindering crack formation. Furthermore, to simulate the actual repeated deformation of flexible electronic skin, a tensile test with 500 cycles was designed on 4 parallel OE-skin samples (Figures 2L and S7A–S7D). As seen, the OE-skin was stretched from its original length of 20 mm to 100% strain. Fatigue occurred mainly during the initial stretching cycles. In the subsequent hundreds of cycles, the tensile force level remained almost unchanged. This is attributed to the highly reversible tensile resistance of the PAAm double-network hydrogel and PU, both of which are elastomeric materials.

Additionally, the OE-skin demonstrated excellent resistance to breaking, such as cutting and puncturing, which was tested with knives and tips (Figures S7E and S7F). Furthermore, the OE-skin was adhered to the flexible knuckle joint of an index finger and then subjected to over 100 impacts using a sharp blade and pointed tip (Video S1). During this process, the OE-skin's surface exhibited temporary yet reversible deformation, indicating a certain level of resistance to cutting and puncturing. As expected, once the blade and tip were removed, the OE-skin quickly returned to its original shape, and the traces of cutting or puncturing disappeared. Subsequently, the OE-skin underwent several dozen bending cycles with the knuckle, maintaining close adhesion to the skin without any mechanical damage, proving its reliability (Video S2). To further quantitatively demonstrate this robustness, we tested the changes in OE-skin's conductivity (*G*), reflection peak (*λ*), and tensile force (*F*) after puncturing it 10 times with a 2 N force, cutting it 10 times with a 2 N force, or putting it through 100 cycles of 100% strain, respectively (Figures S7G–S7I). These changes were compared to the initial values (G_0, λ_0, and F_0), representing the optical, electrical, and mechanical performance of the OE-skin. The values did not show significant decreases, indicating that the OE-skin maintained its stability well under these impacts. Overall, our flexible electronic skin exhibits superior flexibility and robustness, which greatly benefits stable monitoring.

To facilitate wireless control of the behaviors, such as adhesion, of the OE-skin, it was designed to exhibit sensitive and reusable photothermal conversion properties through the incorporation of CNTs, which further induced the temperature-responsive behavior of the hydrogel network (Figures 3A and 3B). Under near-infrared (NIR) irradiation at 808 nm, the temperature of the OE-skin increased due to the photothermal effect, and the rate of temperature rise was modulated by the irradiation power densities (Figure 3C). As seen, lower power densities resulted in a slower temperature rise, affecting the corresponding sensitivity. Conversely, higher power densities led to rapid temperature changes, which were difficult to control and unsafe. A moderate and rapid temperature rise curve was achieved at 0.5 W/cm, which was chosen for subsequent applications. To demonstrate the reproducibility of the OE-skin's photothermal conversion, periodic NIR-induced photothermal cycles were employed (Figure 3D). Through four cycles of pulsed irradiation followed by shutdown, OE-skin completed four heating-cooling cycles. The temperature changes remained sensitive throughout, with no significant deviations during these cycles. This demonstration proves the robust and flexible NIR responsiveness of the OE-skin, facilitating on-demand control.

The thermosensitive hydrogel network of the OE-skin can undergo transitions under photothermal effects. Specifically, the non-covalent component of the double-network hydrogel and gelatin can achieve reversible crosslinked-uncrosslinked transitions under temperature influence (Figures 3A and 3E). Rheological tests indicate that the storage modulus (G′) and loss modulus (G′′) of the isolated gelatin network decrease in response to temperature rise, intersecting at near body surface temperature. This is due to the temperature-reversible effect on the crosslinked hydrogen bonds within the gelatin network. The adhesive hemisphere array formed by the PNIPAm component also responds to temperature.

Higher temperatures can induce changes to its hydrophilic-hydrophobic balance, leading to volume phase transition and subsequent shrinkage. The process was recorded via the mass change rate against temperature (Figure 3F). This behavior exhibited a significant shrinkage rate and a change range around body surface temperature. To visually reflect the phenomenon, fluorescence microscopy was used to observe the NIR-controlled hemispherical array adhered to the substrate (Figure 3G). Meanwhile, the change in fluorescent area was plotted (Figure 3H). In the initial state, the dyed hemispherical array appeared uniformly circular. When NIR irradiation was applied, the hemispheres shrank in volume, reducing the contact area with the substrate. When the NIR was turned off, the hemispheres returned to their original shape, demonstrating their outstanding reversible controllability.

Moreover, broken OE-skin can self-heal under NIR illumination. Besides its excellent mechanical properties, the OE-skin's capability to restore its original shape and performance even after breaking is attractive for flexible electronic devices (Figure S8A). Upon cutting, NIR irradiation causes gelatin molecules to responsively diffuse at the fractured interface and re-establish topological adhesion, allowing the hydrogel network to recover (Figure S8B). Due to the reversibility of hydrogen bonds in gelatin, this self-healing process is also reversible. The mechanical strength and electrical conductivity of the OE-skin were tested before and after multiple healing cycles. The mechanical strength showed partial recovery (Figure S8C), while the electrical conductivity almost fully recovered (Figure S8D).

The OE-skin, profiting from its controllability under NIR, can achieve strong and on-demand wet adhesion (Figure 4A). To induce firm adhesion, the OE-skin was attached to a wet tissue surface and applied pre-pressure after NIR heating. This adhesion relies on three factors. First, the water on the wet tissue surface is trapped in the gaps of the hemispherical array, forming a liquid seal against the internal vacuum (Figure S10). This prevents interfacial water from affecting adhesion, which is a major cause of failure in most tissue adhesives. Second, the negative pressure inside the hemispherical array induces physical contact with the tissue surface, enhancing adhesion. Third, components of the hydrogel network, especially gelatin, diffuse across the tissue interface during NIR heating, forming entanglements. These molecules are more crosslinked by hydrogen bonds when cooling down to body surface temperature, creating topological adhesion. When detachment of the OE-skin is needed, NIR is applied to deactivate all of the above three adhesion factors. The reversible crosslinking of molecules like gelatin is weakened, leading to disentanglement of the hydrogel network from the tissue surface. The shrinkage of the hemispherical array reduces the effective contact area. As the OE-skin is peeled off, interfacial water is released from the cavities, further weakening the adhesion at the interface.

To quantitatively demonstrate the adhesion capability of the OE-skin on tissue surfaces, it was adhered to wet pig skin and compared with a plain patch without the adhesive array (Figure 4B). In tensile and shearing adhesion tests, the samples were stretched vertically and horizontally against the pig skin until complete separation. In both test modes, the OE-skin with hemispherical adhesive arrays exhibited greater stress, indicating stronger adhesion. Meanwhile, greater displacement represented a larger separation distance. Additionally, the arrays resulted in larger curve integration, indicating that more energy was required for interface separation. Furthermore, a systematic quantitative analysis of the adhesion capabilities of the "array" and "plain" groups under different conditions was conducted in terms of strength and energy (Figure 4C). The OE-skin showed the best adhesion on dry skin, with tensile strength sufficient to withstand 1,500 times the OE-skin's own weight, fully meeting the usage requirements. The plain patch also performed well on dry skin, demonstrating the effectiveness of molecular adhesion strategies. However, as previously mentioned, interfacial water on wet skin destroys adhesion. Due to the adhesive array's ability to trap interfacial water, it prevented significant decreases in both strength and energy ($\sim$84% for tensile strength and $\sim$63% for adhesion energy), mitigating the adverse effects of interfacial water. In contrast, the plain patch failed due to interfacial water (Figure 4D).

Furthermore, due to the repeatability of the OE-skin's adhesion mechanism, it can switch between adhesive and non-adhesive states over several cycles, achieving reversible adhesion. After five cycles, its adhesive state still maintains a tensile strength above 25 kPa, while the non-adhesive state has a strength of around 5 kPa, making it easier to peel off (Figure 4E). Repeated testing in the fifth cycle showed that the non-adhesive state had significantly lower stress, displacement, and curve area, with maximum values that were $\sim$22% of the adhesive state, achieving reversible on-demand adhesion (Figure 4F). This feature can be used to adjust position, reuse, and recycle.

We further simulated five cycles of adhesion and repositioning of the OE-skin on human skin. During these cycles, the adhesion remained robust and did not cause any discomfort to the skin (Figure S9A). The optical and electrical properties of the OE-skin were also consistently stable, with a relative stability rate of around 100% (Figure S9B). These experiments demonstrate that repeated removal and adhesion do not affect the normal *in vivo* functioning of the OE-skin. On a theoretical level, reversible adhesion does not impact the functional performance of the OE-skin. Firstly, adhesion is an interfacial behavior that occurs at the part in contact with the skin. The execution of the OE-skin's optical and electrical properties primarily relies on the substrate. Secondly, the intrinsic changes that mediate adhesion are reversible; specifically, the thermosensitive interactions between gelatin and PNIPAm molecules are hydrogen bonds, which are recognized for their reversibility. In particular, adhesion to hairy skin surfaces is also demonstrated in Figure S9 and Videos S1 and S2. The skin adhered to by the OE-skin has fine hair, illustrating its resistance to hair interference. Of course, for areas heavily covered with bushy hair, such as the head, we can refer to clinically recommended skin preparation methods. By removing a small area of hair and cleaning the skin immediately before wearing the OE-skin, we can achieve more stable adhesion and performance, which is both practical and harmless.

To test the on-demand adhesion performance of the OE-skin in practical scenarios, it was adhered to the back of nude mice, allowing the nude mice to move freely for 1 h (Figure 4G). During this period, the OE-skin remained firmly attached without any loosening or displacement. Representative movement of nude mice with the OE-skin affixed to their skin was recorded in Video S3. The nude mice were placed on a rotating disk, where they were freely engaged in various activities such as crawling, running, and twisting. Notably, there were no signs of mice biting or scratching at the OE-skin, confirming that the OE-skin not only adheres robustly but also ensures the comfort of living organisms without affecting their normal behavior. For detachment, NIR was used to heat the OE-skin to 45°C, after which it could be easily removed from the skin with tweezers. Intriguingly, due to the universality of the physical and molecular adhesion mechanisms, the OE-skin achieved firm adhesion on various moist biological tissues, such as muscle, stomach, intestines, and heart (Figures 4H and 4I), indicating its broad potential for monitoring internal tissues. Additionally, it adhered well to various materials like glass, silicon wafers, plastic, rubber, paper, and metal (Figures 4J and 4K), even exhibiting stronger adhesion than on biological samples, which suggests its potential as a sensor in a wider range of applications. As for the potential application scenarios of the OE-skin on non-biological surfaces, in the use of flexible electronic devices, there are instances where direct contact with the body surface is not possible or allowed, such as with divers, patients with surface coverings, or other special populations. In these cases, the substrates to which the OE-skin needs to be adhered might be rubberized diving suits or the plastic surface of dressings. Furthermore, silicon-based or metal-based materials are promising directions for advanced usage in the future, such as in robots, and it is valuable for flexible electronic devices to monitor the temperature or relative motion between two silicon or metal parts. Additionally, the OE-skin is expected to be utilized for the transfer of various samples, such as silicon and glass wafers, with monitoring services to support industrial production.

When employing flexible electronics, issues such as the accumulation of body heat and sweat may also arise. These factors not only affect the accuracy of measurements but also impact the health and safety of living organisms, necessitating properties of breathability and water resistance. For the OE-skin, multiple effects ensure that it is unaffected by sweat. Firstly, the part of the OE-skin that comes into contact with the skin is made of a hydrogel material. As a porous polymer network, breathability is one of the inherent attributes of hydrogels. As demonstrated in Figure S4 , the hydrogel material used in the OE-skin possesses a microscopic porous structure. To further illustrate this, we simulated sweat with droplets applied to the non-array area of the OE-skin (Figure S10A). It could be observed that the liquid quickly penetrated via the hydrogel network. Moreover, the patterned design in the OE-skin could further serve as a chamber to accommodate excess surface liquids, reinforcing the aforementioned effect (Figure S10B). In terms of application, the OE-skin exhibited a high tolerance to liquids, such as water. We adhered the OE-skin to human skin and demonstrated its performance under extremely liquid-rich conditions (submerged in water)

www.the-innovation.org

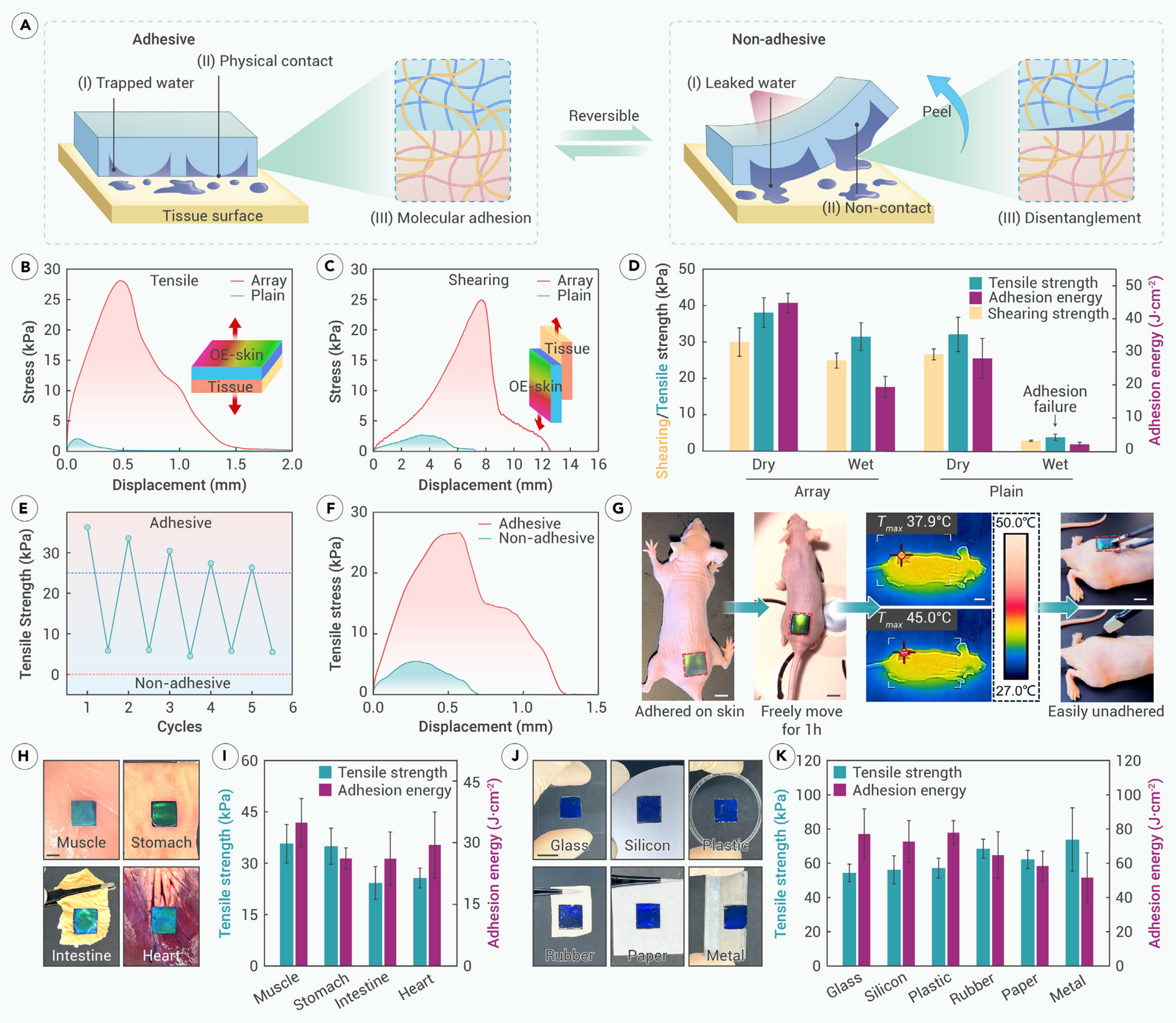

Figure 4. On-demand adhesion of OE-skin (A) The firm adhesion mechanism of OE-skin relies on three factors: trapped interfacial water, physical contact, and molecular adhesion. Under NIR control, all three factors are destroyed, and the OE-skin transforms into a non-adhesive state. (B and C) Adhesion curves of the "array" group with adhesive array and the "plain" group without array on wet skin under tensile and shearing modes. (D) Adhesion strength and energy of array and plain groups on dry and wet skin. The plain group showed obvious adhesion failure on wet skin. $n = 3$. (E) Adhesion cycle curve. The OE-skin is controlled to switch between two states multiple times on demand. (F) Comparison of tensile curves in adhesive and non-adhesive states. (G) Physical picture of adhesion on the skin of moving nude mice and on-demand removal. (H and I) Physical pictures and statistical graphs of adhesion on various biological tissue surfaces. $n = 3$. (J and K) Physical pictures and statistical pictures attached to the surfaces of various materials. $n = 3$. All scale bars: 1 cm.

(Video S4). Such extreme conditions far exceeded the range of human perspiration, and under normal circumstances, the performance of the OE-skin was sufficiently stable.

To meet the requirement of interaction with the human body, the OE-skin is designed with excellent biocompatibility. The CNTs, PU, and hydrogel matrix in this system are all suitable biomedical materials. To verify the cytocompatibility, fibroblast cells were co-cultured with the OE-skin for 3 days according to ISO 10993 standards. Live/dead-cell staining showed live cells in green and dead cells in red. Fluorescence images indicated that cells in both the OE-skin and control groups continued to proliferate over 3 days, with the vast majority remaining viable (Figure 5A). The cells exhibited clear contours and healthy morphology, with few dead cells observed. Further, the quantitative results from the CCK-8 showed no statistical difference in cell viability between the OE-skin and control groups over 3 days, confirming its cytocompatibility (Figure 5B). As a potential implantable device, its blood compatibility was also evaluated through *in vitro* hemolysis tests. The OE-skin interacted with resuspended blood cells, showing no significant hemolysis even at a concentration of 100 mg mL^{-1}, well below the 5% threshold set by ISO 10993 for blood-contacting medical devices (Figure 5C), demonstrating its safety in interactions with blood components.

The *in vivo* compatibility of the OE-skin with tissue surfaces was further evaluated. It was also adhered to the back skin of nude mice. After 1 h of adhesion, NIR was used to trigger OE-skin separation, with commercial tape as a control (Figure 5D). Clinically, medical adhesive-related skin injury (MARSI) caused by commercial tape is common. Initial images showed that the skin of the nude mice remained soft and healthy. However, after adhesion with commercial tape, the skin showed signs of dryness and redness due to the strong but uncontrollable adhesion force and potential irritants, such as organic solvents. In contrast, the OE-skin, due to its biocompatible components and soft tissue-like wet matrix, did not irritate the skin, which remained healthy post-adhesion. Histological analysis of *in situ* skin samples using hematoxylin and eosin (H&E)

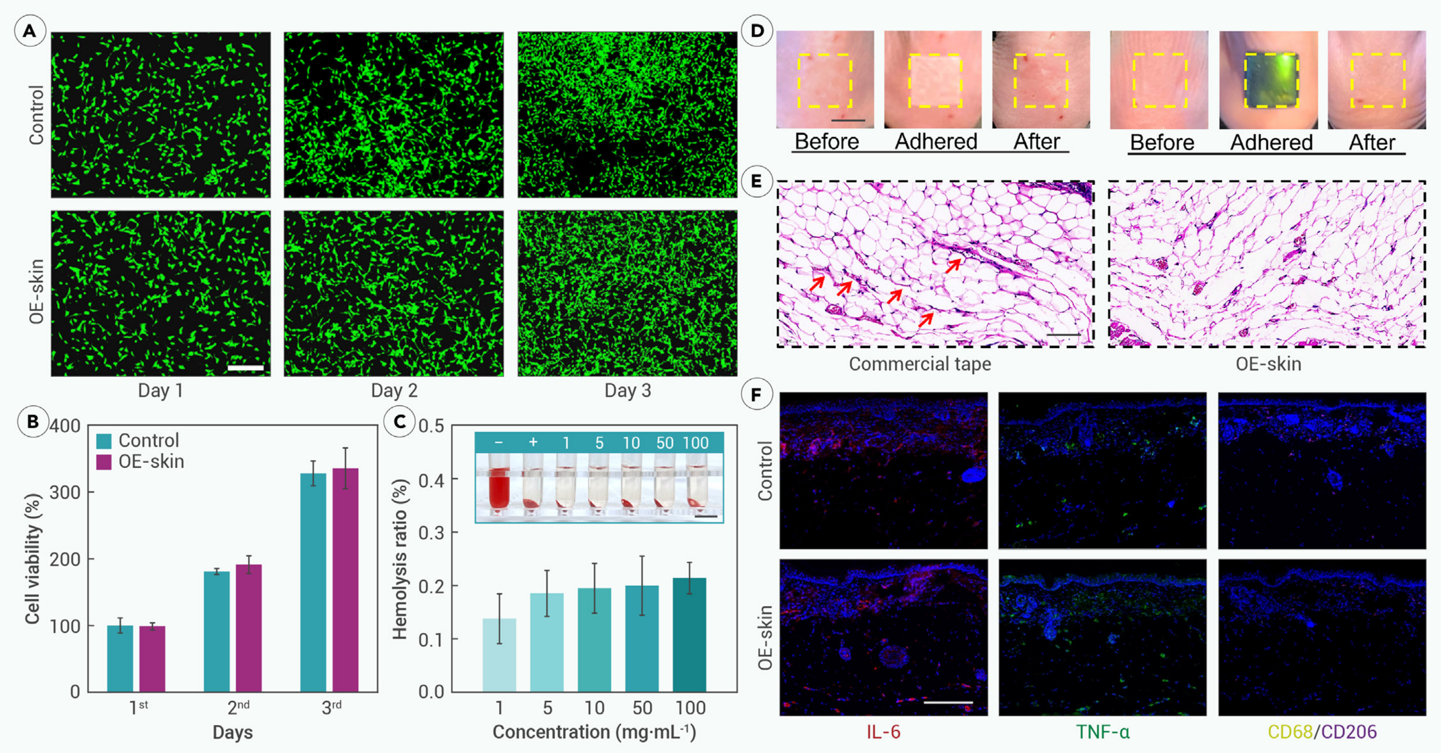

Figure 5. Biocompatibility of OE-skin (A) Fluorescence images show that after 3 days of co-culture, the cells treated with OE-skin and the control group have normal morphology and continue to proliferate. (B) CCK-8 statistical results show that there is no statistical difference in cell viability between the OE-skin group and the control group within 3 days of co-culture. n = 6. (C) Hemolysis rate of different proportions of OE-skin. n = 6. Even at 100 mg mL^{-1}, it is less than the ISO standard of 5%. (D) Physical images of OE-skin and commercial tape adhesion on the skin surface of nude mice. Commercial tape caused adverse reactions, such as redness and swelling, while the skin treated with OE-skin remained healthy. (E) Corresponding histological H&E staining image. Commercial tape caused subcutaneous hemorrhage, as shown by the arrow. (F) OE-skin has good tissue compatibility. IL-6 (red), TNF-α (green), and CD68 (yellow)/CD206 (purple) immunofluorescence staining are shown. Scale bars: 100 μm in (A) and (E), 1 cm in (C) and (D), and 200 μm in (F).

staining (Figure 5E) revealed that commercial tape caused some subcutaneous capillary rupture, with red blood cells observed in the tissue gaps, as shown by the arrows. In contrast, the skin treated with the OE-skin remained intact, with no vascular rupture, allergy, or inflammation observed.

The long-term effects of the OE-skin on the biological surface were further studied and evaluated using various inflammation and immune markers. Specifically, the OE-skin was worn on the delicate skin of nude mice for 6 h. Subsequently, we selected interleukin (IL)-6, tumor necrosis factor alpha (TNF-α), and CD68/CD206 for immunofluorescence staining to demonstrate the tissue compatibility of the OE-skin (Figure 5F). IL-6 and TNF-α are potent pro-inflammatory cytokines that play a key role in the acute phase response, and changes in their levels can indicate whether there is an inflammatory response to the OE-skin. CD68 is commonly used as a marker for macrophages, while CD206 is a specific marker for M2-type macrophages. Fewer fluorescent areas were observed in both the blank control group and the OE-skin group, indicating that, from the perspective of multiple markers, the OE-skin caused rare inflammation and immune responses of biological tissue, with no macrophage infiltration, demonstrating good biocompatibility. Furthermore, the relative fluorescence-positive area of the aforementioned markers was statistically analyzed in Figure S11. For all indicators, there was no statistical difference between the OE-skin groups and the blank control groups, indicating that the long-term action of the OE-skin did not affect the health of the biological body surface. These results confirm the OE-skin's biocompatibility and suitability for practical applications at the cellular, tissue, and organism levels.

The OE-skin possesses multimodal flexible monitoring capabilities, both optical and electrical, allowing it to independently reflect various important physiological information such as temperature and motion (Figure 6A). Specifically, the hydrogel layer functions as an electrical sensor, while the PU film serves as an optical sensor. The conductivity of the hydrogel layer primarily arises from the free electrons in the CNTs and their uniform dispersion within the hydrogel network, which establishes electron transfer pathways. When the motion or temperature changes, the resistance of the OE-skin changes accordingly, allowing for the monitoring of physiological signals. When motion induces strain, the original electron transfer pathways in the OE-skin expand, leading to a sensitive increase in resistance, which is directly reflected by a decrease in the brightness of the light-emitting diode (LED) light in series (Figure 6B). On the other hand, when the temperature rises, the carrier mobility of the CNTs in the OE-skin increases, resulting in a sensitive decrease in resistance and an increase in LED brightness (Figure 6C). The opposite is also true. Thus, it is capable of monitoring both temperature and motion. However, the isolated electrical signal cannot decouple the two physiological signals, which can be further resolved by the optical sensor.

With regard to the optical sensor PU film, as previously mentioned, the unique structural color of the PU opal film arises from its nanoscale photonic band-gap effect. According to Bragg's law, when the OE-skin undergoes stretching during motion, the normal interplanar spacing d decreases, leading to a change in structural color. During stretching, the structural color of the OE-skin changes continuously and noticeably from red to blue-violet (Figure 6B). However, the interplanar spacing d of the PU film is not sensitive to temperature changes within the measurement range. Additionally, since the material composition remains unchanged, the structural color of the OE-skin remains stable with temperature variations (Figure 6C). This allowed the structural color change to primarily reflect motion signals, thereby decoupling the electrical signal. Consequently, this dual-modal sensing can sensitively and accurately respond independently to both temperature and motion over a wide range. Such performance can serve many practical application scenarios. For instance, in the case of athletes, their target tissues are often in motion, and real-time monitoring of the amplitude and speed of movement can yield valuable insights. Concurrently, the temperature of the target tissues is also important. A temperature that is too low may indicate hypothermia or poor circulation, which may occur during outdoor activities. On the other hand, a high body temperature may signal conditions such as inflammation or heatstroke. In such cases, the OE-skin could guide athletes in maintaining their body temperature within a safe range without compromising the completion of their intended exercises. Additionally, for some patients, it is necessary to monitor both mobility and temperature in their tissues. In this context, temperature can indicate pathological conditions such as insufficient blood supply or inflammation.

www.the-innovation.org

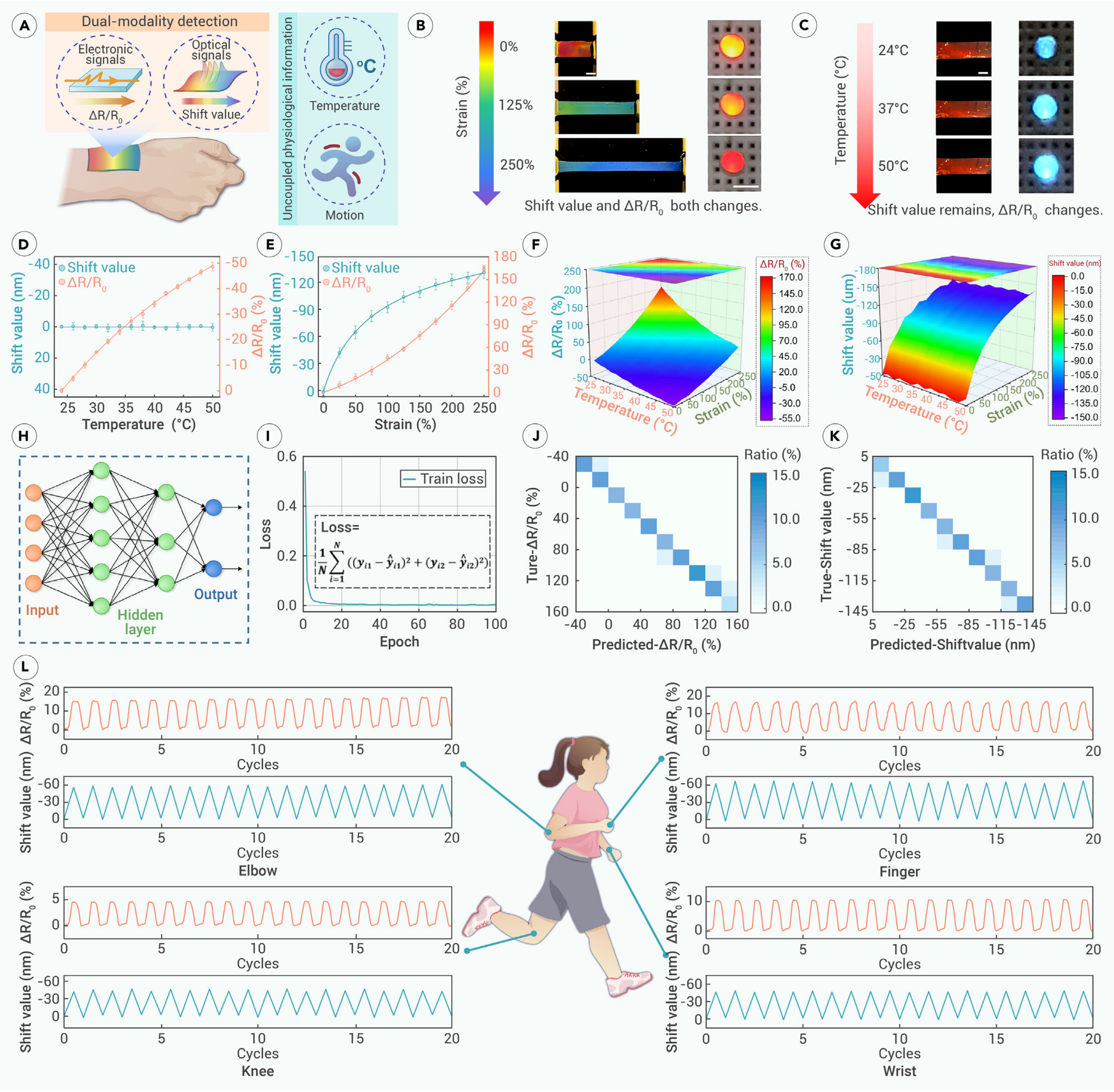

Figure 6. Dual-modal monitoring capabilities of OE-skin (A) OE-skin obtains decoupled temperature and motion information through optical and electrical dual-modal sensing. (B and C) Physical images of OE-skin's color and resistance changes under different strains (B) and temperatures (C), respectively, displaying sensitive responses. (D and E) The response of the OE-skin's optical and electronic signal to temperature (D) and strain (E), respectively. *n* = 3. (F and G) Electrical (F) and optical (G) signals under dual-variable responses of temperature and strain. The signals are stable with a wide range. (H) Neural network structure used to assist OE-skin regression analysis of variable relationships. (I) The loss function of the neural network is close to saturation in 100 epochs, proving that the network model has well captured the variable relationship of OE-skin. The loss is the covariance between the network prediction value and the theoretical value. (J and K) Heatmap distribution of the network prediction value and the actual value. The values are concentrated on the diagonal line, proving that the consistency is strong. (L) OE-skin collects 20 cycles of motion information at the elbow, fingers, knee, and wrist. All scale bars: 0.5 cm.

The dual-modal sensing capabilities of the OE-skin were further systematically tested. Within the isolated temperature changing range of 24°C–50°C, the shift value of its structural color reflection peak showed no significant fluctuation, remaining around 0 nm. However, the resistance changing rate ($\Delta R/R_0$) gradually decreased to 48.6% as the temperature increased (Figure 6D). For motion information, when the strain independently changed from 0% to 250%, the shift value of the OE-skin changed from 0 to −133.7 nm, confirming the apparent blue shift of the structural color. Meanwhile, $\Delta R/R_0$ sensitively increased to 163.4%, indicating an increase in resistance due to strain (Figure 6E). It is worth mentioning that the monitoring range of the OE-skin's response to temperature and strain fully covers the range of human physiological signals. The optical and electrical signal outputs of the OE-skin were studied under the dual effects of temperature and strain within the aforementioned range. For $\Delta R/R_0$, influenced by both electron transfer pathways and mobility, the highest resistance value was observed at low temperature and high strain, and vice versa (Figure 6F). The optical signal shift value remained largely insensitive to temperature under different strains, showing an overall blue-shift trend with increasing strain (Figure 6G). Both 3D surfaces exhibited a wide range of values and were relatively smooth, with no

significant noise or fluctuations, demonstrating the sensitivity and stability of the OE-skin's output signals.

To better establish a model between variables, a neural-network-based machine learning algorithm was used to perform regression and prediction on the input and output signals collected by the OE-skin. The network includes input and output as well as two hidden layers (Figure 6H). The variable data, consisting of signals with two input values (temperature and strain) and two output values ($\Delta R/R_0$ and shift value), were divided into a training set (~66.7%) and a test set (~33.3%). During the 100 epochs of training, the network's loss function gradually decreased and eventually became saturated (Figure 6I), indicating a good fit between the optical and electrical signals collected by the OE-skin and the network model, effectively capturing their relationship. In testing, the predicted values of the neural network were compared with the actual values. They showed excellent uniformity. The heatmap displayed data concentrated near the diagonal in all divided intervals, confirming the close proximity of the predicted and actual values, demonstrating decent accuracy (Figures 6J and 6K). This suggests that the OE-skin can be combined with advanced methods like neural networks to better facilitate human-machine interactions.

Additionally, OE-skin can meet the needs of fully wireless electronic skin in a simple and cost-effective manner. Figure S13 illustrates a simulated circuit diagram for the wireless scenario. In this configuration, the OE-skin is connected in series with a miniaturized power source (such as a button battery with a diameter of less than 5 mm) and a simple wireless transmission component, eliminating the need for bulky current meters or similar devices. The aforementioned wireless transmission components, such as buzzers, LED light sources, or other Bluetooth, Wi-Fi, and other wireless modules, can all be within a few millimeters in size and can be worn directly on the human body. At this point, the OE-skin's electrical signals are easily converted into wireless acoustic volume, optical brightness, or other radio signals, thus rendering it wireless. For example, Figures 6B and 6C demonstrate its ability to drive a tandem LED light with a 3 V voltage source, with the brightness directly reflecting changes in the resistance of the OE-skin. The reflection peaks of structural colors can also be read wirelessly. We designed the color change range in the visible light spectrum, facilitating direct observation with the naked eye (Figures 6B and 6C). Furthermore, the color of specific pixel blocks can also be recorded through a camera and converted into wavelength information using formulas. We believe that this wireless design can be further complemented by artificial intelligence methods, as shown in Figures 6H–6K, thereby endowing the OE-skin with more accurate and convenient usage methods.

The practicality of the OE-skin, benefiting from dual-modal signal monitoring, on-demand adhesion, and biocompatibility, was validated in various scenarios. The OE-skin was adhered to the skin of various joints in the human body. For example, when applied to the wrist, it could monitor stable signals over more than 100 motion cycles (Figure S12). The entire monitoring process was continuous and precise, without any breakage or loosening of the OE-skin. Detailed displays of 20 motion cycles detected on the fingers, wrist, elbow, and knee all yielded stable and reliable signals (Figure 6L). Video S5 further demonstrates the OE-skin's activity along the most flexible joints of the human body, such as finger joints. These positive demonstrations indicated that the proposed OE-skin is promising as a new generation of flexible platforms for precise, stable, and wide-range multimodal biological information sensing.

CONCLUSION

In summary, inspired by natural biology, we designed and integrated a conductive adhesive hydrogel with a PU inverse opal film to develop a novel flexible OE-skin with on-demand adhesion and synergistic opto-electronic sensing functions through decoupled visual changes. The device exhibits vibrant structural colors due to the inverse opal structure of PU. Additionally, the presence of CNTs endows the device with sensitive electrical sensing. Using responsive biomimetic hemispherical arrays and molecular adhesion, the OE-skin achieves firm, on-demand, and reusable adhesion, which can be controlled by NIR irradiation for intelligent manipulation. The mechanical properties and biocompatibility of the OE-skin further lay a foundation for its robustness and safety in *in vivo* applications. Moreover, as a dual-modal flexible device, the OE-skin can reflect decoupled body temperature and motion information in real time through optical and electrical signals. The *in vitro* and *in vivo* results demonstrate that the OE-skin has great potential in the field.

For the technological advancement and commercialization potential of the OE-skin, the materials used, such as PU, PAAm, PNIPAm, and gelatin, are highly industrialized, offering low cost and easy accessibility. The manufacturing process does not rely on overly complex production machinery. We have validated the reproducibility and stability of the preparation process under laboratory conditions. In the future, we can further optimize preparation conditions, produce larger micro-nano templates, and cut them post-molding to prepare more OE-skin products at once, better meeting the demands of mass production. From the perspective of integration with existing technologies, we have explored its interaction capabilities with neural networks. It can be further combined with existing signal acquisition, transmission, and storage methods, for example, coupling the device with Bluetooth modules for wireless electrical signal collection or integrating it with smartphone camera modules to directly read spectra visually. Additionally, current technologies allow the development of mobile applications for online storage and analysis of the data collected by OE-skin. Hence, the OE-skin might offer new possibilities for intelligent bio-flexible sensing for vision-based physiological monitoring, medical diagnosis, human-computer interactions, and more.

DATA AND CODE AVAILABILITY

All data are available in the main text or the supplemental information.

REFERENCES

1. Jiang, Y., Zhang, Z., Wang, Y.X. et al. (2022). Topological supramolecular network enabled high-conductivity, stretchable organic bioelectronics. *Science* **375**:1411–1417. DOI:https://doi.org/10.1126/science.abj7564.
2. Zheng, Y., Michalek, L., Liu, Q. et al. (2023). Environmentally stable and stretchable polymer electronics enabled by surface-tethered nanostructured molecular-level protection. *Nat. Nanotechnol.* **18**:1175–1184. DOI:https://doi.org/10.1038/s41565-023-01418-y.
3. Wang, W., Jiang, Y., Zhong, D. et al. (2023). Neuromorphic sensorimotor loop embodied by monolithically integrated, low-voltage, soft e-skin. *Science* **380**:735–742. DOI:https://doi.org/10.1126/science.ade0086.
4. Madhvapathy, S.R., Wang, J.J., Wang, H. et al. (2023). Implantable bioelectronic systems for early detection of kidney transplant rejection. *Science* **381**:1105–1112. DOI:https://doi.org/10.1126/science.adh7726.
5. Zhang, T., Liu, N., Xu, J. et al. (2023). Flexible electronics for cardiovascular healthcare monitoring. *Innovation* **4**:100485. DOI:https://doi.org/10.1016/j.xinn.2023.100485.
6. Zhong, D., Wu, C., Jiang, Y. et al. (2024). High-speed and large-scale intrinsically stretchable integrated circuits. *Nature* **627**:313–320. DOI:https://doi.org/10.1038/s41586-024-07096-7.
7. Kim, S.H., Basir, A., Avila, R. et al. (2024). Strain-invariant stretchable radio-frequency electronics. *Nature* **629**:1047–1054. DOI:https://doi.org/10.1038/s41586-024-07383-3.
8. Zhang, B., Li, J., Zhou, J. et al. (2024). A three-dimensional liquid diode for soft, integrated permeable electronics. *Nature* **628**:84–92. DOI:https://doi.org/10.1038/s41586-024-07161-1.
9. Reeder, J.T., Xie, Z., Yang, Q. et al. (2022). Soft, bioresorbable coolers for reversible conduction block of peripheral nerves. *Science* **377**:109–115. DOI:https://doi.org/10.1126/science.abl8532.
10. Yuk, H., Wu, J. and Zhao, X. (2022). Hydrogel interfaces for merging humans and machines. *Nat. Rev. Mater.* **7**:935–952. DOI:https://doi.org/10.1038/s41578-022-00483-4.
11. Yuk, H., Lu, B. and Zhao, X. (2019). Hydrogel bioelectronics. *Chem. Soc. Rev.* **48**:1642–1667. DOI:https://doi.org/10.1039/C8CS00595H.
12. Wang, C., Chen, X., Wang, L. et al. (2022). Bioadhesive ultrasound for long-term continuous imaging of diverse organs. *Science* **377**:517–523. DOI:https://doi.org/10.1126/science.abo2542.
13. Deng, S., Li, Y., Li, S. et al. (2024). A multifunctional flexible sensor based on PI-MXene/SrTiO3 hybrid aerogel for tactile perception. *Innovation* **5**:100596. DOI:https://doi.org/10.1016/j.xinn.2024.100596.
14. Baik, S., Lee, H.J., Kim, D.W. et al. (2019). Bioinspired adhesive architectures: from skin patch to integrated bioelectronics. *Adv. Mater.* **31**:1803309. DOI:https://doi.org/10.1002/adma.201803309.
15. Fan, H. and Gong, J.P. (2021). Bioinspired underwater adhesives. *Adv. Mater.* **33**:2102983. DOI:https://doi.org/10.1002/adma.202102983.
16. Lee, J.H., Cho, K. and Kim, J.K. (2024). Age of flexible electronics: emerging trends in soft multifunctional sensors. *Adv. Mater.* **36**:2310505. DOI:https://doi.org/10.1002/adma.202310505.
17. Gong, S., Lu, Y., Yin, J. et al. (2024). Materials-Driven Soft Wearable Bioelectronics for Connected Healthcare. *Chem. Rev.* **124**:455–553. DOI:https://doi.org/10.1021/acs.chemrev.3c00502.
18. Yuk, H., Varela, C.E., Nabzdyk, C.S. et al. (2019). Dry double-sided tape for adhesion of wet tissues and devices. *Nature* **575**:169–174. DOI:https://doi.org/10.1038/s41586-019-1710-5.
19. Yang, J., Bai, R. and Suo, Z. (2018). Topological adhesion of wet materials. *Adv. Mater.* **30**:1800671. DOI:https://doi.org/10.1002/adma.201800671.

20. Ma, Z., Bao, G. and Li, J. (2021). Multifaceted design and emerging applications of tissue adhesives. *Adv. Mater.* **33**:2007663. DOI:https://doi.org/10.1002/adma.202007663.
21. Nam, S. and Mooney, D. (2021). Polymeric Tissue Adhesives. *Chem. Rev.* **121**:11336–11384. DOI:https://doi.org/10.1021/acs.chemrev.0c00798.
22. Li, W., Yang, X., Lai, P. et al. (2022). Bio-inspired adhesive hydrogel for biomedicine—principles and design strategies. *Smart Med.* **1**:e20220024. DOI:https://doi.org/10.1002/SMMD.20220024.
23. Du, Z., Qiao, F., Tong, L. et al. (2024). Mimicking Mytilus edulis foot protein: A versatile strategy for robust biomedical coatings. *Innovation* **5**:100671. DOI:https://doi.org/10.1016/j.xinn.2024.100671.
24. Nan, K., Feig, V.R., Ying, B. et al. (2022). Mucosa-interfacing electronics. *Nat. Rev. Mater.* **7**:908–925. DOI:https://doi.org/10.1038/s41578-022-00477-2.
25. Wang, C., Shirzaei Sani, E., Shih, C.D. et al. (2024). Wound management materials and technologies from bench to bedside and beyond. *Nat. Rev. Mater.* **9**:550–566. DOI:https://doi.org/10.1038/s41578-024-00693-y.
26. Zhao, C., Park, J., Root, S.E. et al. (2024). Skin-inspired soft bioelectronic materials, devices and systems. *Nat. Rev. Bioeng.* **2**:671–690. DOI:https://doi.org/10.1038/s44222-024-00194-1.
27. Frey, S.T., Haque, A.B.M.T., Tutika, R. et al. (2022). Octopus-inspired adhesive skins for intelligent and rapidly switchable underwater adhesion. *Sci. Adv.* **8**:eabq1905. DOI:https://doi.org/10.1126/sciadv.abq1905.
28. Packard, A. (1961). Sucker display of octopus. *Nature* **190**:736–737. DOI:https://doi.org/10.1038/190736a0.
29. Lee, H., Um, D.S., Lee, Y. et al. (2016). Octopus-inspired smart adhesive pads for transfer printing of semiconducting nanomembranes. *Adv. Mater.* **28**:7457–7465. DOI:https://doi.org/10.1002/adma.201601407.
30. Baik, S., Kim, D.W., Park, Y. et al. (2017). A wet-tolerant adhesive patch inspired by protuberances in suction cups of octopi. *Nature* **546**:396–400. DOI:https://doi.org/10.1038/nature22382.
31. Kong, B., Liu, R., Cheng, Y. et al. (2022). Structural color medical patch with surface dual-properties of wet bioadhesion and slipperiness. *Adv. Sci.* **9**:2203096. DOI:https://doi.org/10.1002/advs.202203096.
32. Wang, Y., Shang, L., Chen, G. et al. (2020). Bioinspired structural color patch with anisotropic surface adhesion. *Sci. Adv.* **6**:eaax8258. DOI:https://doi.org/10.1126/sciadv.aax8258.
33. Fu, F., Shang, L., Chen, Z. et al. (2018). Bioinspired living structural color hydrogels. *Sci. Robot.* **3**:eaar8580. DOI:https://doi.org/10.1126/scirobotics.aar8580.
34. Liu, F., Deswal, S., Christou, A. et al. (2022). Neuro-inspired electronic skin for robots. *Sci. Robot.* **7**:eabl7344. DOI:https://doi.org/10.1126/scirobotics.abl7344.
35. Shih, B., Shah, D., Li, J. et al. (2020). Electronic skins and machine learning forintelligent soft robots. *Sci. Robot.* **5**:eaaz9239. DOI:https://doi.org/10.1126/scirobotics.aaz9239.

ACKNOWLEDGMENTS

This work was supported by the National Key Research and Development Program of China (2022YFB4700100), the National Natural Science Foundation of China (T2225003, 52073060, 81930048, 82330061, and 61927805), the Nanjing Medical Science and Technique Development Foundation (ZKX21019), the Local Innovative and Research Teams Project of Guangdong Pearl River Talents Program (2019BT02X105), the Hong Kong Research Grant Council General Research Fund (15217721 and 15125724), the Shenzhen Science and Technology Innovation Commission (JCYJ20220818100202005), and the Hong Kong Polytechnic University Fund (P0045680, P0043485, P0045762, and P0049101).

AUTHOR CONTRIBUTIONS

Y.Z. conceived the idea and designed the experiment; W.L., J.L., and X.D. conducted the experiments and data analysis; W.L. and P.L. wrote the manuscript; W.S., Q.T., and P.L. participated in the discussion of the results; and all authors were engaged in the manuscript proofreading.

DECLARATION OF INTERESTS

The authors declare no competing interests.

SUPPLEMENTAL INFORMATION

It can be found online at https://doi.org/10.1016/j.xinn.2025.100877.

The Innovation, Volume *6*

Supplemental Information

Multi-bioinspired electronic skins with on-demand adhesion and opto-electronic synergistic display capabilities

Wenzhao Li, Jinbo Li, Xiaoya Ding, Qitao Tan, Weijian Sun, Puxiang Lai, and Yuanjin Zhao

Supplemental information

Multi-bioinspired electronic skins with on-demand adhesion and opto-electronic synergistic display capabilities

Wenzhao Li [1,2], Jinbo Li [3], Xiaoya Ding [3], Qitao Tan[2, 4], Weijian Sun [1,5,*], Puxiang Lai [2, 4, 6, *], Yuanjin Zhao[1,3,*]

[1] Department of Rheumatology and Immunology, Nanjing Drum Tower Hospital, School of Biological Science and Medical Engineering, Southeast University, Nanjing 210096, China

[2] Department of Biomedical Engineering, The Hong Kong Polytechnic University, Hong Kong SAR 999077, China

[3] Wenzhou Institute, University of Chinese Academy of Sciences, Wenzhou, Zhejiang 325001, China

[4] Research Institute for Sports Science and Technology, The Hong Kong Polytechnic University, Hong Kong SAR 999077, China

[5] Department of Gastrointestinal Surgery, The First Affiliated Hospital, Wenzhou Medical University, Wenzhou 325035, China

[6] Joint Research Center for Biosensing and Precision Theranostics, The Hong Kong Polytechnic University, Hong Kong SAR 999077, China

Correspondence: fame198288@126.com (W. S.); puxiang.lai@polyu.eu.hk (P. L.); yjzhao@seu.edu.cn (Y. Z.)

1. Experimental section

Mechanical performance test

In the tensile tests, the tension started from the original length to break. While in the cyclic tests, strains of hydrogels were cycled between 0-100% for tension, for a total of 500 cycles. Breaking strength and strain are defined as the strain and strain at break.

Stress is the ratio of tension to the original cross-sectional area and strain is the ratio of elongation to original length.

Photothermal performance demonstration

The OE-skin was cut into 2×2 cm^2 size and adhered to pig skin. It was irradiated with 808 nm NIR laser of the same shape and size as the OE-skin. The power density of NIR is defined as the ratio of power to spot area. The OE-skin was irradiated with 0.25, 0.5, 0.75 and 1 $W\cdot cm^{-2}$ NIR. The temperature was recorded. In the photothermal cycling experiment, NIR was switched between 3 min on and 1 min off, repeating 4 cycles.

Shrinking and recovering of the hemispherical array

The hemispherical array was heterogeneously loaded with the fluorescent dye Rhodamine B. The OE-skin was attached to a transparent substrate. The hemispherical fluorescent area was recorded as S_0. Then the OE-skin was heated to 45°C using NIR and kept for 1 min. At this time, the hemispherical structure shrunk and separated from the substrate. The fluorescent area S was recorded again. The NIR was turned off, the shape of the hemispherical array was restored, and the area S was recorded again. The contraction rate was equal to S/S_0. The area S was measured by ImageJ.

Adhesion experiments

OE-skin was attached to pig skin, heated to 45°C using NIR and maintained at a pre-pressure of 10 kPa for 1 min, which was recorded as the adhesive state. When detached on demand, NIR was used to heat up to 45°C and maintained for 1 minute until the adhesion failed, which was recorded as the non-adhesive state. In tensile and shearing adhesion tests, separation was performed from the directions perpendicular and parallel to the OE-skin respectively until the adhesion broke. At this time, stress was recorded as the ratio of adhesion force to adhesion area. Strength is the maximum value of stress. Adhesion energy is the energy required for interface separation in

tensile adhesion experiments, which is the integral of adhesion force and displacement image area.

In Vitro cytocompatibility assay

3T3 cells were planted in 96-well plates as 1500 per well. The control group was cultured with DMEM medium, and the experimental group was cultured with 0.2 $g \cdot mL^{-1}$ OE-skin according to ISO 10993. The cells were incubated in a carbon dioxide incubator at 37°C for 72 h. The cells were tested according to the instructions of CCK-8 and live/dead staining kit at 24 h, 48 h and 72 h.

In Vitro blood compatibility assay

Fresh blood from rats was taken and centrifuged at 3000 rpm for 10 minutes to purify red blood cells. The blood was resuspended in 1×PBS and the above process was repeated three times. Finally, a red blood cell suspension (1% v/v) was prepared using PBS. 1, 5, 10, 50 and 100 $mg \cdot mL^{-1}$ of OE-skin were incubated with the above red blood cell suspension at 37 °C for 3 hours. Afterwards, the resulting liquid was centrifuged at 3000 rpm for 10 minutes to remove the precipitate. The absorbance in the supernatant was then measured within 540 nm. Red blood cells interacting with deionized water were used as positive controls and recorded as a hemolysis rate of 100%. Red blood cell suspensions in PBS were used as negative controls and recorded as a hemolysis rate of 0%.

In vivo animal experiment

Six male BALB/C nude mice aged 6 weeks were selected. They were adhered to the back of the nude mice. The adhesion and detachment processes were the same as above. They were allowed to move freely for one hour during adhesion. Commercial tape was used as a control and the adhesion was also maintained for one hour. The final state picture was taken ten minutes after detachment. The *in-situ* skin samples were subjected to histological analysis using H&E staining. All animal experiments were conducted in strict accordance with the recommendations in the Guide to the

Care and Use of Experimental Animals. The Animal Experiment Ethics Committee of Wenzhou Institute of University of Chinese Academy of Sciences reviewed and approved all animal care and experimental protocols. The approval number is WIUCAS24081502.

Physiological information monitoring

For the response curve of optical and electrical signals to temperature, OE-skin was attached to the surface of pig skin, and then the pig skin was placed on a hot plate to heat it, and the changes in resistance and spectral reflection peaks were recorded. For the response curve of optical and electrical signals to strain, the patch was attached to the regular rectangular Ecoflex surface to simulate biological tissue. Ecoflex has higher elasticity than pig skin, and pig skin will undergo irreversible deformation under large strains, affecting the experiment. The resistance and reflection peak values were read as they were stretched. The resistance value in the initial state was recorded as R_0, and the change was recorded as ΔR. The change in the reflection peak was recorded as shift value. For repeated motion monitoring, the patch was attached to the index finger, wrist, elbow and knee joints, and recorded continuously. All resistances were recorded using a desktop digital multimeter. The spectrum was recorded using a spectrometer. All data sampling points were read after stabilization.

Signal processing of OE-skin by neural network

A fully connected neural network (FCNN) model was used to perform regression analysis on the relationship between temperature, strain, ΔR/R0 and shift value. The model consists of an input layer, a hidden layer and an output layer, and information flows unidirectionally from the input layer to the output layer. The input layer contains four neurons, representing the four input variables mentioned above. The hidden layer contains two layers, the first hidden layer contains five neurons, and the second hidden layer contains three neurons. These layers are fully connected, that is, each neuron in each layer is connected to each neuron in the next layer. The output

layer contains two neurons, representing the two target variables to be predicted. Before training the neural network, the input data is normalized. Each data point contains four variables, and a total of 600 variables were collected, generating 150 data points. Randomly divide two-thirds of these data points into training sets and the rest into test sets. The epoch is 100 iterations. Loss function based on the mean square error is used to measure the difference between the predicted value and the actual value.

Characterization

The micro- and nanostructures were observed by SEM (SU8010, HITACHI, Japan). The observed optical and fluorescence microscopic images were obtained by stereomicroscopy (Olympus BX51, Tokyo, Japan) and inverted fluorescence microscopy (ZEISS Axio Vert. A1, Germany). Micro-CT images were obtained by micro-computed tomography (SkyScan 1176, Bruker, Germany). Rheological properties were tested by a rotational TA rheometer (DHR-2). Electrical resistance was tested by a benchtop digital multimeter (DMM6500, Keithley, USA). The absorption spectrum of the solution was measured by a multi-mode microplate reader (Varioskan LUX, ThermoFisher, USA). Mechanical testing was performed with an electronic universal material testing machine (5944, Instron, USA). 808 nm NIR was generated by a laser (DS3-808 nm -10 W BWT China). The temperature was recorded by an infrared thermometer (E5-XT, FLIR, USA). The reflectance spectra were taken by an optical microscope (Olympus, BX51) equipped with a fiber optic spectrometer (Ocean Optics, USB2000-FLG).

Statistical analysis

Unless specifically mentioned, the whole data were expressed as mean ± standard deviations in multiple repeated groups. The standard deviation is represented by the error bars. For direct comparisons between two groups, Student's t-test was employed. When more than two groups were involved, one-way ANOVA was utilized. The differences were considered statistically significant if $*p < 0.05$, $**p < 0.01$, $***p <$

0.001 or ****p < 0.0001. Sample size (n) is detailed in the specific figure legends. All statistical analysis was conducted using SPSS software.

2. Supplemental Figures

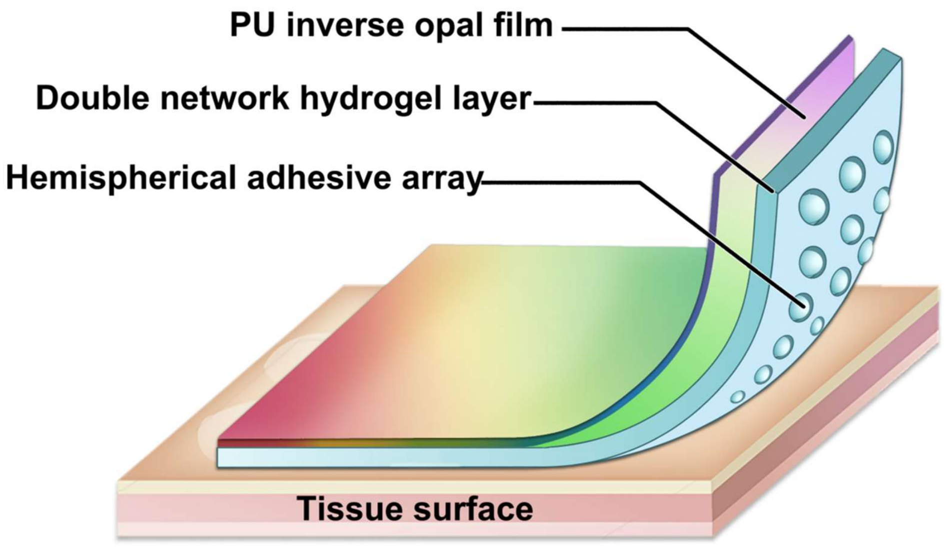

Figure S1. OE-skin includes PU film and hydrogel layer. There is an adhesive array on the hydrogel layer.

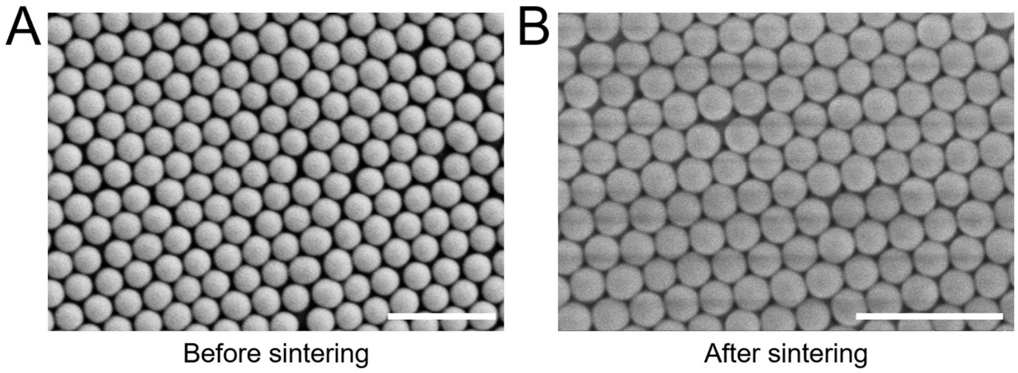

Figure S2. Comparison of SiO_2 template before and after sintering. The particles are more closely spaced after sintering. (A) Before sintering. (B) After sintering. Scale bars are 1 μm.

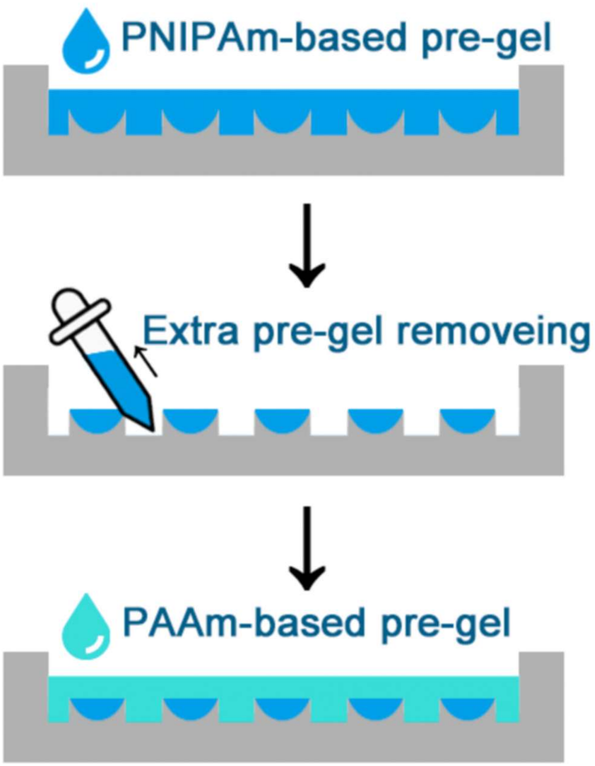

Figure S3. Detailed schematic diagram of the stepwise template infusion method. Two pre-gel solutions of hydrogels were infused separately and cross-linked to obtain a heterogeneous structure.

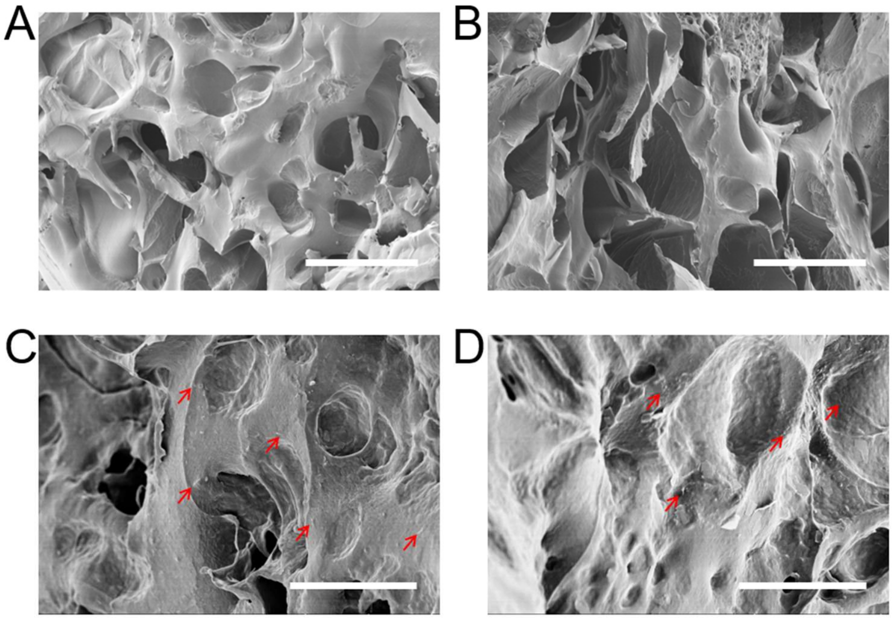

Figure S4. SEM images of hydrogel pores. (A-B) PAAm (left) and PNIPAm (right) double network hydrogels have rich porous structures. (C-D) CNTs are dispersed in the pores of PAAm (left) and PNIPAm (right) double network hydrogels. Scale bars are 100 μm in (A) and (B), as well as 10 μm in (C) and (D).

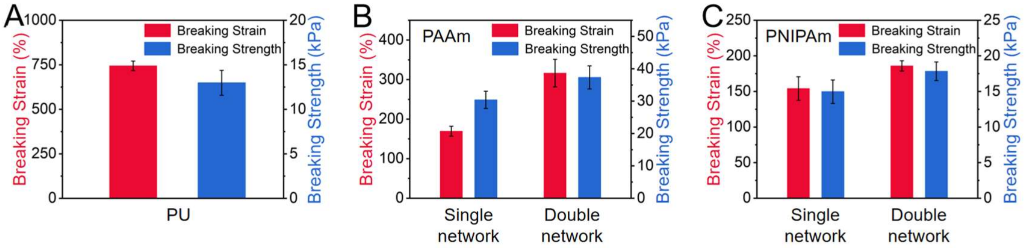

Figure S5. The mechanical properties of PU film, single and double network hydrogels. (A) PU. (B) Single and double network PAAm. (C) Single and double network PNIPAm. Double network hydrogels generally have better mechanical properties. n=3 for each statistic.

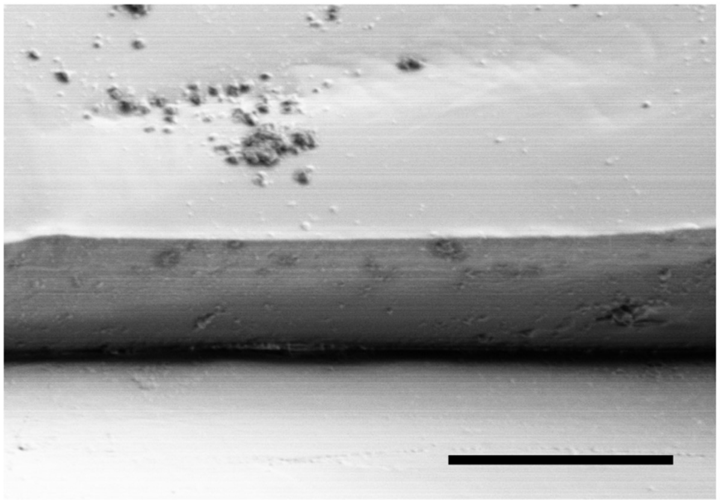

Figure S6. The interface between PU film and hydrogel layer is tightly coupled. The scale bar is 20 μm.

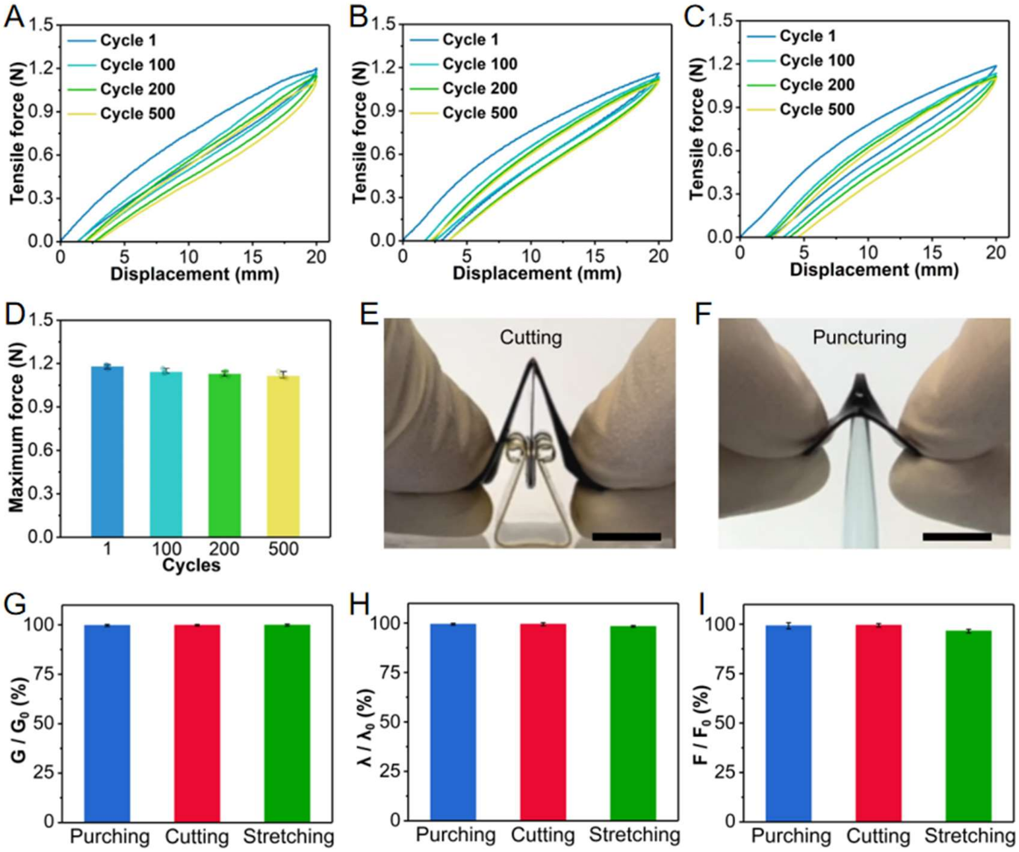

Figure S7. Mechanical robustness of OE-skin. (A-D) OE-skin has mechanical robustness in cyclic tensile tests. Additional parallel experiments are in (A-C), and statistical analysis of the maximum tensile force is in (D), n=4. (E-F) OE-skin has good anti-breaking performance. The cutting test is in (E), and the puncturing test is in (F). Scale bars are 1 cm. (G-I) The electrical, optical, and mechanical performance of OE-skin after puncturing, cutting, and cyclic stretching. The changes in conductivity (G), reflection peak (λ), and tensile force (F) are shown in (G), (H), and (I), respectively, n=3.

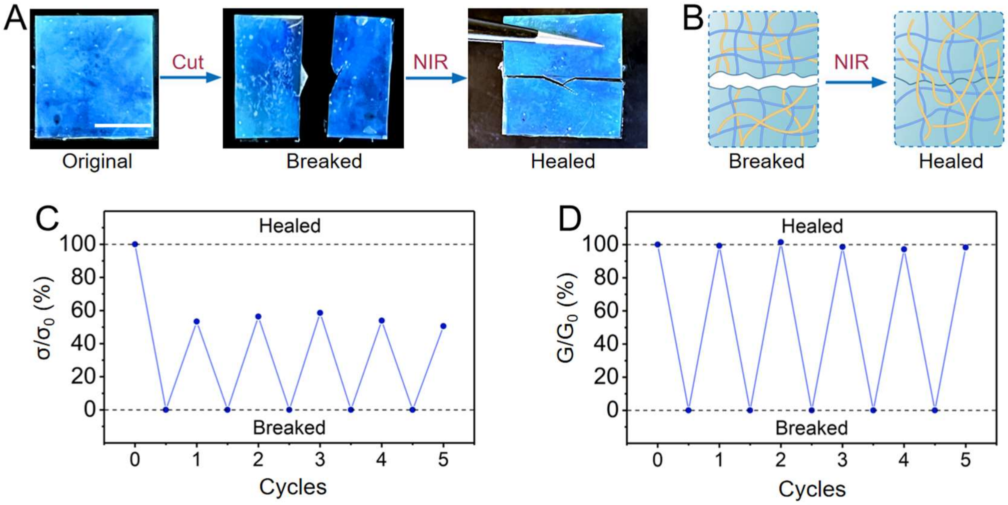

Figure S8. NIR-controlled healing properties of OE-skin. (A) Physical images of the OE-skin being broken and repaired by NIR. (B) Schematic diagram of the healing process. (C) Changes in fracture strength σ during in five breaked-healed cycles. (D) Corresponding conductivity G changes. Scale bars are 1 cm.

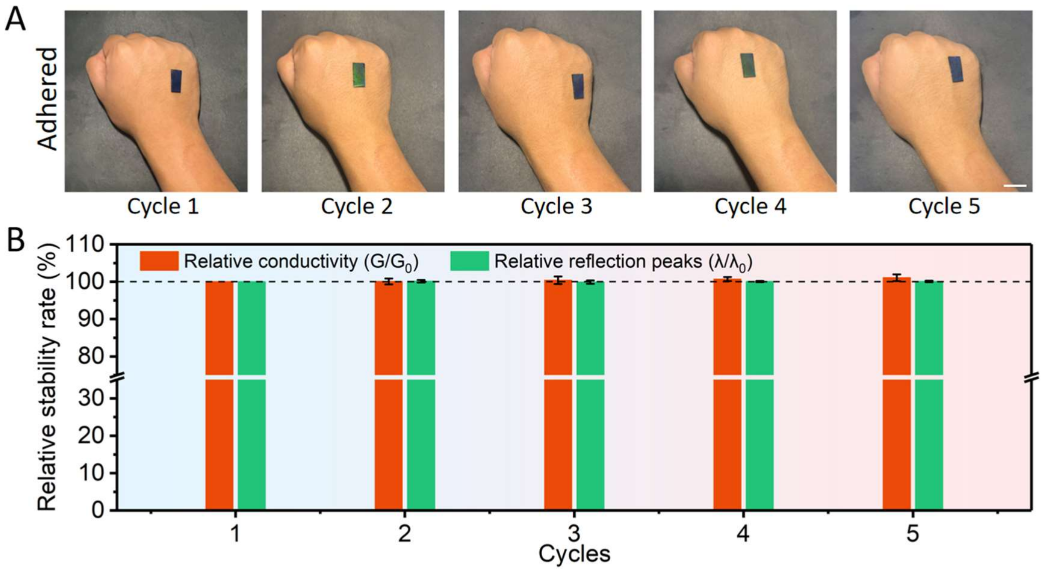

Figure S9. *In vivo* adhesion cycles and corresponding performance stability. (A) Five cycles of adhesion and repositioning of OE-skin on the human skin. The adhesion remained robust without any discomfort. The scale bar is 1 cm. (B) The optical and electrical properties during 5 cycles.

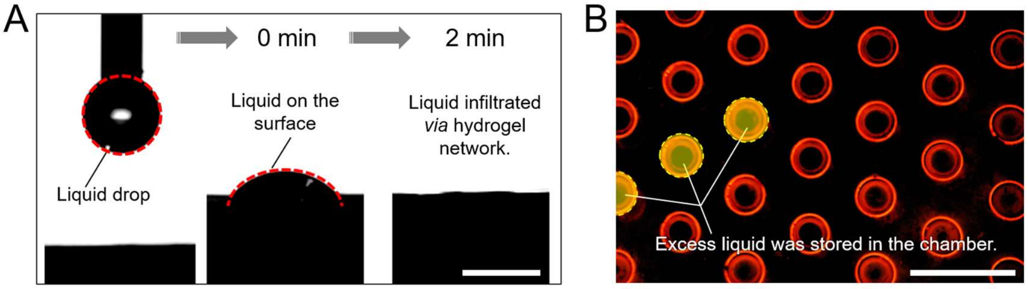

Figure S10. Permeability and water resistance of OE-skin. (A) Droplets used to simulate sweat were applied to the non-array area of OE-skin. The droplets quickly penetrated the hydrogel network within 2 minutes, demonstrating the permeability of OE-skin to sweat. (B) Fluorescence image of interfacial water trapped by the adhesive array. The interfacial water is dyed red by fluorescent dye. Scale bars are 1 mm in (A) and 2.5 mm in (B).

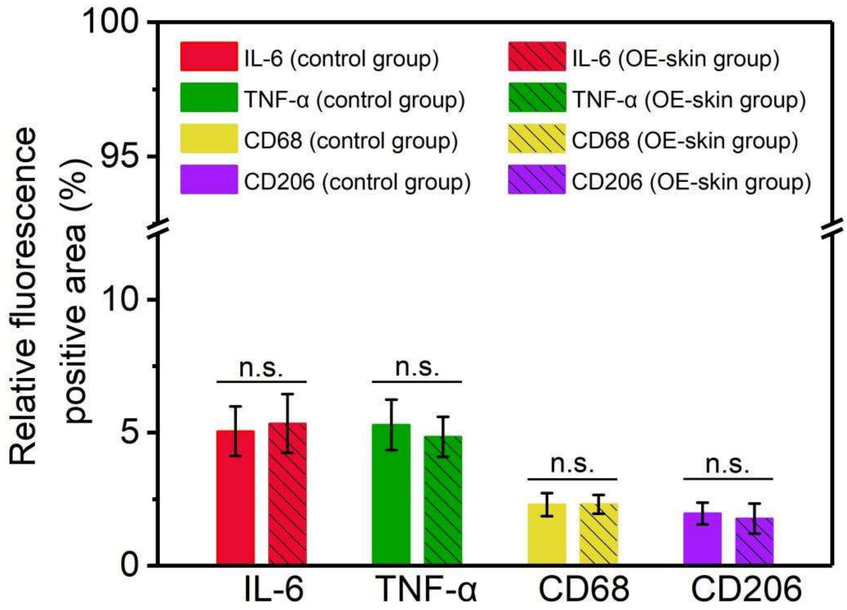

Figure S11. Relative fluorescence positive area in immunofluorescence sections of inflammation and immunity markers. There was no statistical difference between OE-skin groups and the control group, further indicating that the long-term tissue compatibility of OE-skin.

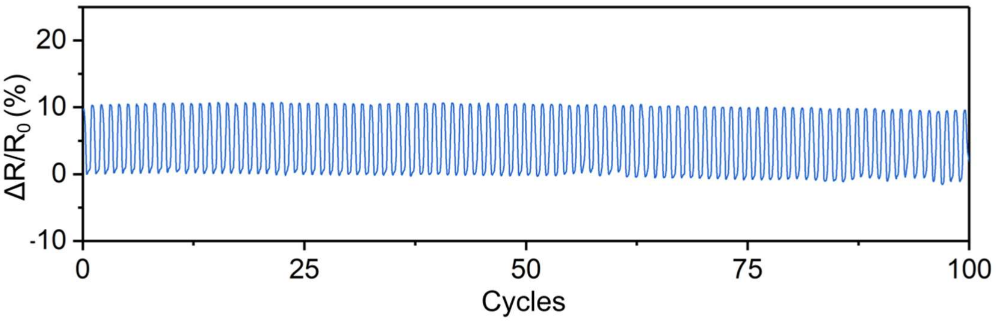

Figure S12. OE-skin collects 100 cycles of motion signals from the wrist.

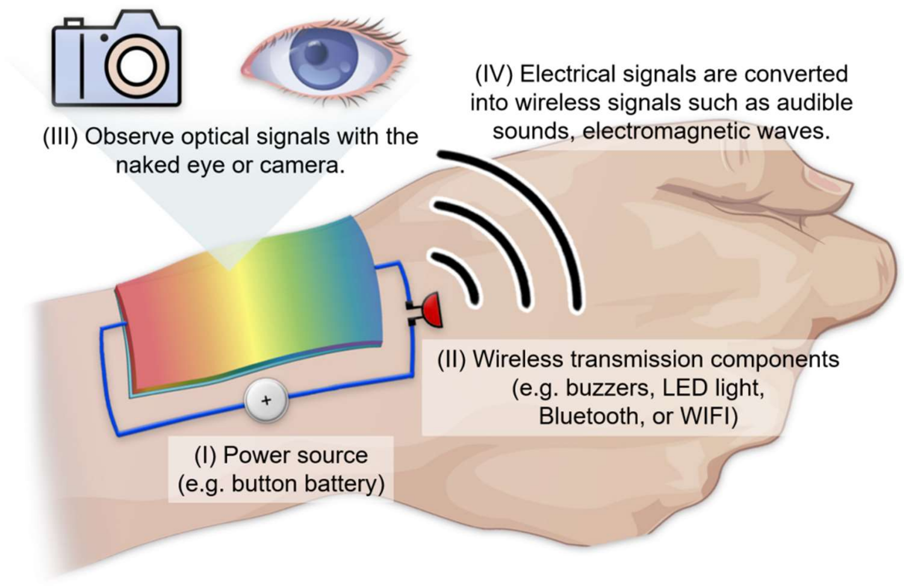

Figure S13. The integration of OE-skin into a fully wireless electronic skin is illustrated in a simple and cost-effective manner. (I) Miniaturized power source. (II) Miniaturized wireless transmission components. (III) Optical signals can be observed visually. (IV) Electrical signals (resistance changes) are converted into other wireless signals, such as audible sound and electromagnetic waves. Audible sound can be directly perceived by the human ear.
